# The host genotype actively shapes its microbiome across generations in laboratory mice

**DOI:** 10.1186/s40168-024-01954-2

**Published:** 2024-12-05

**Authors:** Laurentiu Benga, Anna Rehm, Christina Gougoula, Philipp Westhoff, Thorsten Wachtmeister, W. Peter M. Benten, Eva Engelhardt, Andreas P. M. Weber, Karl Köhrer, Martin Sager, Stefan Janssen

**Affiliations:** 1 Central Unit for Animal Research and Animal Welfare Affairs, Medical Faculty and University Hospital Düsseldorf, Heinrich Heine University Düsseldorf, Düsseldorf, Germany; 2 Algorithmic Bioinformatics, Justus Liebig University Giessen, Giessen, Germany; 3 Cluster of Excellence on Plant Science, Institute of Plant Biochemistry, Heinrich Heine University Düsseldorf, Düsseldorf, Germany; 4 Genomics and Transcriptomics Laboratory, Biological and Medical Research Center, Heinrich Heine University Düsseldorf, Düsseldorf, Germany

**Keywords:** Microbiota, Microbiota standardization, Metagenome, Intestinal, Littermates, Mouse, Genotype, Maternal legacy, Transmission, Skin

## Abstract

**Background:**

The microbiome greatly affects health and wellbeing. Evolutionarily, it is doubtful that a host would rely on chance alone to pass on microbial colonization to its offspring. However, the literature currently offers only limited evidence regarding two alternative hypotheses: active microbial shaping by host genetic factors or transmission of a microbial maternal legacy.

**Results:**

To further dissect the influence of host genetics and maternal inheritance, we collected two-cell stage embryos from two representative wild types, C57BL6/J and BALB/c, and transferred a mixture of both genotype embryos into hybrid recipient mice to be inoculated by an identical microbiome at birth.

**Conclusions:**

Observing the offspring for six generations unequivocally emphasizes the impact of host genetic factors over maternal legacy in constant environments, akin to murine laboratory experiments. Interestingly, maternal legacy solely controlled the microbiome in the first offspring generation. However, current evidence supporting maternal legacy has not extended beyond this initial generation, resolving the aforementioned debate.

Video Abstract

**Graphical Abstract:**

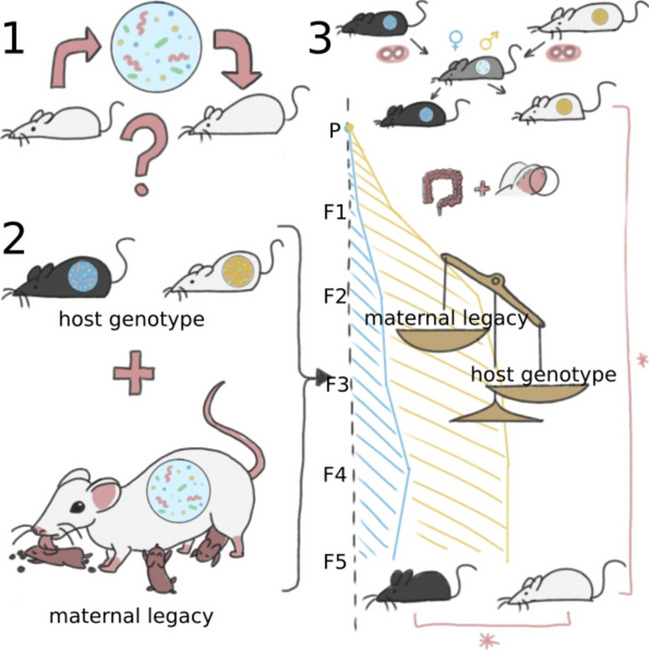

**Supplementary Information:**

The online version contains supplementary material available at 10.1186/s40168-024-01954-2.

## Introduction

The human body is colonized by as many microbial cells as there are human cells [1]. Research of the last decades revealed the enormous impact of the microbiome on human health and wellbeing (see [2] for a review). Numerous factors have been identified that modulate the human-colonizing microbiome like diet, exercise, and animal contact (see [3] for a review). It is the combination of the host’s genotype and its microbiome that together drive the host’s phenotype [4].

Understanding the mechanisms controlling the formation and function of microbial communities is essential in human biology. Standardization of endogenous and exogenous variables, such as genotype and environmental factors, is hardly achievable in larger experimental animal models and impossible in humans. Therefore, mice are consecrated as the most used laboratory animals due to their advantages for experimental work. They served in deciphering fundamental physiological and pathological aspects in mammals. Available murine models range in complexity from simplified microbial communities, such as “Schaedler flora” [5], “altered Schaedler flora” [6], GM15 [7], Oligo-Mouse-Microbiota 12 [8], or humanized-microbiome models [9, 10] over specified pathogen-free (SPF) laboratory mice [11], which are altered in a series of biochemical gut parameters [12], immunological [13] or anti-cancer fitness-promoting traits [14], to the more complex, wild mouse microbiota models [15].

From an evolutionary perspective, it seems unlikely that the host leaves microbial composition to chance. Extreme (genetically modified) genotypes affect the functionality of the immune system and thus contribute to changes in the composition of gut microbiota [16–20]. Multiple quantitative trait loci (QTL) from specific genomic regions seem to contribute to the host tailoring of the microbiome [21–24]. Two factors, among other undefined loci, are the major histocompatibility complex (MHC; H-2 in mice), as demonstrated by the analyses of bacteria-derived cellular fatty acids [25] and IBD susceptibility-involved genes, such as caspase recruitment domain member 9 (Card9) [26]. Some studies showed genome-wide linkage with abundances of specific microbial taxa such as *Lactobacillus* [21, 27] or *Faecalibacterium prausnitzii* [28], whereas others document the influence of the “host genotype” and the environment on the whole microbiome [29–31].

Researchers aware of the importance of the microbiome in the experimental work proposed to scientific journal editors a mandatory documentation of all factors that may influence the microbiome, such as host genotype, husbandry details, or experimental methods [4]. Factors like diet, bedding material, drugs, cage mates, or ventilation are relatively easy to control for. The control of factors, which we subsume as maternal legacy, like passage through the birth canal, weaning, coprophagy, and grooming is almost impossible or implies a significant increase in resources. However, they are known to impact the microbiome and thus most likely impact the host phenotype [19, 31, 32]. One could even speculate that maternal legacy alone is the evolutionary process to vertically transmit a defined microbiome to offspring generations.

The central question remains open, namely which of the two factors maternal legacy or host genotype contribute (more) to the active shaping of a host’s microbiome?

A practical implication could be that strain differences from mice of alternative vendors would harmonize under identical environmental conditions through cross-fostering if maternal legacy was to dominate microbial composition.

Existing literature is inconclusive about effect sizes of maternal legacy vs. host genotype. The vendor and genetic background, in terms of host genotype, seem to influence murine gut microbiota [33]. Nevertheless, studies using embryo transplantation and litter cross-fostering in mouse rearing and housing document that rather environmental conditions and maternal legacy exert a dominant contribution in shaping microbiota composition. A drift of the microbiota to a host genotype and facility-specific composition seem to occur under the influence of these factors [34]. Also, authors of [35] assumed that the foster mother’s gut microbiota rather than the host genotype influence gut microbiota composition in early life [36], whereas the study of Korach-Rechtman et al. (2019) indicates dominance of host genotype over the maternal inoculation by cross-breeding experiments [31]. Overall, authors of [37] account for the host genotype less than 20% of the gut microbiota variation in mice, whereas the findings of [38] suggest that in humans, the gut microbiome and host genotype are largely independent.

Numerous rodent studies that conclude on the influence of host genotype were performed either in immune defective phenotypes or were drawn secondarily to the main goals of the respective studies, often on highly related mice, which were purchased from commercial vendors shortly before the beginning of the respective study. In addition, no natural course of microbiota colonization and transmission over the generations was followed; rather, artificial colonization with/or in association to antibiotics treatment was performed [39]. Moreover, most studies exclusively focused on the gut microbiome. Only recently, pioneering studies regarding the influence of host genotype on the microbiome of other body sites such as the skin [40] and respiratory tract [41, 42] have been conducted in human and murine lung [43], while surveys, e.g., the genital tract, are still missing.

To disentangle the factors host genotype and maternal legacy, we here obtained presumably microbial free two-cell stage embryos of two representative wild types, namely C57BL6/J (B6J, *n* = 42) and BALB/c (C, *n* = 57), and transferred a mix of embryos into six SPF hybrid recipient mice (RM), which were generated from B6J dams and C sires. Therefore, offspring started from the same microbiome, acquired through maternal legacy of RM.

We continued the experiment over five generations of separated breeding while minimizing impact of environmental factors through housing in individually ventilated cages (IVC). For reference, we also sampled six SPF mice of each host genotype independently obtained from our mouse facility (Duesseldorf, Germany), housed in open cages instead of IVC, and bought from a commercial vendor (Janvier, France).

To further dissect maternal legacy, we implemented three cage lineages per host genotype, i.e., strict inbreed lineages that never came in contact in the following generations. We applied 16S rRNA gene sequencing of colon content and the skin of the ear to obtain microbiome profiles of 334 mice in total. It was shown for immunodeficient mice that the host genotype itself alters the microbiome and leads to profound metabolome systemic and not just local effects within the gut [44]. For systemic insights, we therefore collected blood serum to obtain metabolomic data.

Our data show that under controlled environmental factors, host genotype is the driving factor in microbiome composition over multiple generations in inbred laboratory mice. However, the maternal legacy effect is non-negatable, especially in earlier generations. Our analysis also documents a host genotype-dependent increase of particular pathobiont microorganisms such as *Akkermansia muciniphila*, as well as host genotype-specific metabolome correspondence.

## Material and methods

### Mouse strains and husbandry procedures

The mice strains C57BL/6 J (B6J), BALB/c (C), and their F1 hybrid B6CF1 (RM) originated from the specified-pathogen-free (SPF) colony of the Central Unit for Animal Research and Animal Welfare Affairs (ZETT) Duesseldorf. They were free of all agents listed in Table 3 of the FELASA recommendations for health monitoring of rodents [11] and supplementary of *Staphylococcus aureus*, *Proteus* spp., *Klebsiella* spp., *Bordetella bronchiseptica*, *Bordetella pseudohinzii*, *Pseudomonas aeruginosa*, *Muribacter muris*, and *dermatophytes*. The access to this microbiological unit was restricted to a few animal caretakers through a sit-over barrier system and complete change of clothes with sterile clothes consisting of suit overall, underwear, socks, shoes, face mask, head cover, and gloves. This unit was populated exclusively with mice strains hygienically sanitized by means of embryo transfer. For the experiment, the mice were kept in individually ventilated cages (IVC) filled with Shepherd’s™ ALPHA-dri® bedding sheets (Shepherd Speciality Papers, Kalamazoo, USA) and had access ad libitum to autoclaved rodent chow (Ssniff, Soest, Germany) and acidified water. All cages were located in the same IVC rack during the whole period of the experiment and were housed under 12:12 h light/dark cycles, at a 22 ± 2 °C room temperature and 55 ± 5% humidity. All mice cages were changed weekly with autoclaved fresh cages containing the same bedding, food, and water.

### Study design and sampling

**Fig. 1:**
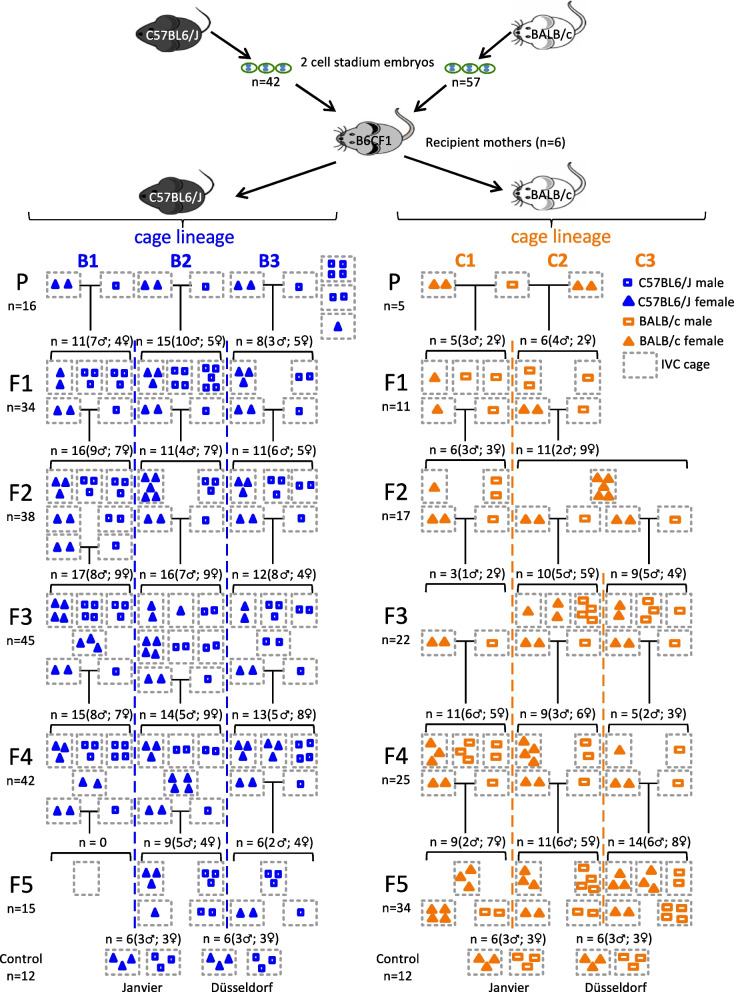
Breeding strategy. We obtained C57BL6/J (B6J, *n* = 42) and BALB/c (C, *n* = 57) two-cell stadium embryos from donor mice. A mix of both host genotype embryos was transferred into six recipient dams of a hybrid host genotype B6CF1, such that each dam gave birth to pups of both host genotypes. Offspring (P generation) was separated by host genotype into six cage lineages (B1–3, C1–3). Inbreeding for generations F1 to F5 always occurred within the same cage lineage (dashed lines). Gray dots indicate individually ventilated cages. Open squares and solid triangles indicate male and female mice, respectively, while blue icons indicate B6J and orange icons C host genotype, respectively. Last row gives numbers for control mice

To obtain B6J and C embryos, female mice were intraperitoneally superovulated using 7 IU PMSG for B6J and 5 IU PMSG for C (Intergonan® 240 IE/mL, MSD Tiergesundheit, Unterschleißheim, Germany) and 7-IU hCG for B6J and 5-IU hCG for C (Predalon® 5000 IE, Essex Pharma GmbH, Waltrop, Germany) 48 h later, followed by mating with males of the same strain. On day 1.5 after hCG administration, embryo donors were sacrificed, their oviducts extracted, and the embryos at the two-cell stage flushed using M2 medium (Sigma-Aldrich, Munich, Germany) according to [45]. An average number of eight two-cell embryos of each B6J and C strain were transferred into the oviduct of each of the six pseudopregnant-related B6CF1-recipient foster mothers (RM) used in this study as described previously [45]. The 6 RM were further placed in 3 individually ventilated cages, each containing 2 of the RM, where they gave birth after approximately 19 days to the mice parental (P) generation consisting of 16 B6J and 5 C mice (Fig. 1). The P mice males and females were weaned at the age of approx. 3 weeks and placed together in two male and two female cages until the age of approx. 7 weeks when they were either used for mating or placed in separate male or female cages until they reached the adult sampling age of 15 weeks when sampling occurred. Three days before mating, dirty bedding originating from the males’ cages was transferred to the respective female’s cage in order to synchronize the ovulation. Three P generation breeding trios of one male and two females were settled for the B6J mice, whereas for the C mice, the only male available was mated for 3 days with two of the C females and then transferred to the second C female cage. For the following generations, a breeding trio was settled from each previous breeding cage, except for the F2 generation of C strain, where two breeding trios were settled from a cage (Fig. 1). The exact cage location and lineage, the sex and the number of mice resulted per host genotype, and generation can be depicted in Fig. 1. In addition, 12 mice each (3 males and 3 females of each B6J and of C strain respectively), originating from Janvier Labs (Le Genest-Saint-Isle, France) and ZETT Duesseldorf respectively, were included as controls. Duesseldorf controls were housed in open cages until sampling. Janvier controls were purchased at an age of 14 weeks and afterwards housed in IVC. The sampling occurred at the age of 15 weeks for all mice, except for a few singular breeding mother mice that still had to nurse for one or two further weeks and reached thus 16 or 17 weeks at sampling. The age of 15 weeks was chosen since at this age the mice display a stable mature gut microbiome [46].

### Sample collection and DNA extraction

To harvest the samples, 15-week-old mice were euthanized by bleeding in narcosis. The collected blood served for sera preparation. Next, approximately 2/3 of the left earlobe and the two to three most distal fecal pellets from the colon were harvested using sterile instruments and used for the analysis of the skin and gut microbiome respectively. All samples were placed into 1.5-mL sterile Eppendorf cups and immediately frozen at -80 °C until further use. All samples were collected between 8:00 and 11:00 a.m. on several days. DNA extraction from colon pellets and skin was performed using the DNeasy PowerSoil and DNeasy Blood & Tissue Kit (Qiagen, Hilden, Germany) respectively using the manufacturer’s protocol. In the final step, DNA was eluted in EB buffer (Qiagen), and the yield was measured by NanoDrop One (Thermo Fisher Scientific, Waltham, USA). Extracted DNA was frozen at -20 °C until further processing.

### 16S amplicon library preparation and sequencing

Genomic DNA samples used for 16S rRNA gene sequencing were quantified by photometric measurement using NanoDrop One device (Thermo Fisher Scientific Inc.). Preparation of the 16S rRNA gene amplicon libraries for the Illumina MiSeq System was performed according to the Illumina 16S metagenomics protocol (part no. 15044223 Rev. B) sequencing the V3–V4 region of the 16S rRNA gene (primers: FWD:CCTACGGGNGGCWGCAG, REV:GACTACHVGGGTATCTAATCC) with the change of the material input to 1 µL of the sample volume. Two Illumina i5 and i7 8-bp barcodes were used for each sample for a 384 multiplexing schema. Final libraries were analyzed for fragment length distribution with the Fragment Analyzer (Agilent Technologies, Inc.) using the HS NGS Fragment Kit (1–6000 bp) assay (DNF-474). Concentrations were determined by fluorometric measurement using the Qubit fluorometer and a DNA high-sensitive assay (Thermo Fisher Scientific Inc.). Libraries were normalized to 2 nM, equimolar pooled, and subsequently sequenced on a MiSeq system (Illumina Inc.) with a read setup of 2 × 301 bp by using a MiSeq Reagent v3 (600-cycle) Kit with three flow cells in total.

### Statistical analysis

The base calling and simultaneous demultiplexing were done via bcl2fastq (v2.19.0.316), and primers were trimmed using cutadapt (v2.10, [47]). Cutadapt removes adapter sequences from high-throughput sequencing reads. Quality-controlled sequence data was imported into the Qiita study management platform (https://qiita.ucsd.edu/, hosted at UC San Diego, [48]) under study ID 13422. Through Qiita, we used QIIME (v1.9.1, [49]) to clip reads to regions above a Phred score of 3, drop reads containing N base calls, and trim reads to 150 bp. The generation of feature tables was performed by de novo amplicon sequence variant (ASV) determination using the Deblur approach (v1.1.0, [50]). Taxonomy for Deblur sequences was assigned via the q2-feature-classifier [51] of QIIME2 (v2023.2, [52]) using the pre-trained Naive-Bayes classifier https://data.qiime2.org/classifiers/greengenes/gg_2022_10_backbone_full_length.nb.qza, which is based on full-length ribosomal sequences of Greengenes2 [53]. As Greengenes2’s taxonomy currently lacks labels for mitochondria and chloroplasts, we classified ASV sequences against the older Greengenes (v13.8, [54]) database specifically ASVs assigned to “c__Chloroplast” or “f__mitochondria” as a pre-filtering. Low biomass skin samples have been controlled against “kitome” contamination [55] through Decontam [56] as suggested [57]. We used Decontam as provided through QIIME2 version amplicon 2024.5 in “combined” mode and a threshold of 0.5.

In the following, the ASV feature table was used to determine the alpha and beta diversities using QIIME2 as well as differential abundance analysis. 

We used q2-fragment-insertion of QIIME2 (v2023.5, [58]) to phylogenetically place all Deblur sequences into the reference Greengenes 13.8 99% identity tree [54] to obtain a phylogeny for downstream phylogenetic aware alpha- and beta-diversity metrics, i.e., Faith’s phylogenetic diversity index [59] and weighted and unweighted UniFrac [60].

### Alpha and beta diversity

We chose a rarefaction depth of 1000 reads per sample for skin samples and 6000 for gut samples by analyzing alpha rarefaction curves for the three metrics “observed_features,” “Shannon,” and “Faith’s PD” using 10 iterations for every depth. These depths were best for representing the highest taxonomic diversity while losing the least number of samples in our dataset. Alpha diversity was calculated using the plain number of observed features (richness), Shannon index, Chao1, and Faith’s phylogenetic diversity index (Faith PD). Beta diversity was calculated using the phylogenetic measure weighted and unweighted UniFrac, as well as the non-phylogenetic measure Bray-Curtis dissimilarity [61] and Jaccard-Needham dissimilarity [62]. Dissimilarity was visualized as principal coordinate analysis (PCoA) in a 3D Emperor plot [63]. Significance between groups in alpha diversity was assessed by two-sided Mann-Whitney-Wilcoxon or Kruskal-Wallis tests and for beta-diversity group significance with PERMANOVA using 9999 permutations, correcting via the Benjamini-Hochberg approach.

### Differential abundance analysis

Statistically significant differentially abundant taxa were identified using analysis of composition of microbiomes (ANCOM) as a QIIME2 plugin [64].

### Joint analysis with Robertson et al. data

We obtained raw read files for [65] from NCBI’s BioProject with accession number PRJEB28381 and trimmed V4 primers 515F (Parada) and 806R (Apprill) off the reads (cutadapt v2.10, [47]). Further downstream processing (e.g., ASV calling, taxonomy assignments, filtering) was done identically to our dataset; see above. As both datasets target different variable regions (V4 and V3–V4 for Robertson et al. and ours, respectively), not a single ASV nucleotide sequence will be shared between both. We therefore limited alpha- and beta-diversity analysis to phylogenetic metrics, which indirectly merged the datasets by phylogenetically placing ASVs into the same Greengenes 13.8 99% identity tree [54]. The joint feature table was rarefied to 6000 reads per sample.

### Metabolome analysis by GC–MS

Ten serum samples from the generations F3 and F4 belonging to each B6J and C host genotype were chosen for GC-MS-based metabolic profiling, following previously established protocols [44]. Metabolite extraction was conducted with minor modifications to the methodology described by [66]. In brief, 1 mL of a -20 °C cooled extraction solution composed of acetonitrile (ACN)/isopropanol (IPA)/water (H_2_O) (3:3:2, v/v/v) was mixed with 30 µL of a 25-µM internal standard (ISTD) solution (ribitol and N,N-dimethylphenylalanine). Then, 20 µL of sample was added to the extraction solution, vortexed for 10 s, shaken for 5 min, and then centrifuged for 2 min at 14,000 rcf at 4 °C. Next, two 450-µL aliquots of the supernatant were transferred to new tubes, and 500 µL of an ice-cold solution of ACN/water (50:50, v/v) was added to remove any excess protein. After additional centrifugation for 2 min at 14,000 rcf, the supernatant was transferred to a pre-cooled tube and dried by vacuum centrifugation.

The dried sample was reconstituted in 150 µL of the extraction solution and dried again via vacuum centrifugation after transfer into a glass vial. The sample was derivatized with methoxyamine hydrochloride and N-methyl-N-(trimethylsilyl) trifluoroacetamide as described in [67]. After incubation for 2 h at room temperature, 1 µL was injected into a GC-MS system (7890A GC and a 5977B MSD, Agilent Technologies), and chromatography was performed as described in [68]. Metabolite identification was performed on two levels. A quality control (QC) sample containing a mixture of target compounds was included as a reference to identify target compounds in the sample based on mass spectra similarity and retention time (annotation level: reference). In addition, the AMDIS software (http://chemdata.nist.gov/mass-spc/amdis/ v2.72, 2014) was used for deconvolution of mass spectra of target peaks before comparing spectra to the NIST14 Mass Spectral Library (https://www.nist.gov/srd/nist-standard-reference-database-1a-v14). Matches with more than 80% mass spectra similarity were assigned accordingly (annotation level: NIST match). Peaks were integrated using the software MassHunter Quantitative (v b08.00, Agilent Technologies). For relative quantification, metabolite peak areas were normalized to the peak area of the internal standard ribitol.

### Triglycerides quantification

Triglycerides in serum were recorded using the colorimetric Triglyceride Quantification Kit (catalog number MAK266, Sigma-Aldrich, Darmstadt, Germany) according to manufacturer’s protocol.

## Results

### The host genotype overrides the maternal legacy effect over generations in constant environments

Conflicting reports about the dominance of host genotype or maternal legacy on microbial composition, and the suggestion of Robertson and co-authors [65] to first generate F2 littermates for maximal microbial homogeneity before conducting genotype-phenotype experiments, let us set out our breeding experiment in which we followed microbial composition of two commonly used wild-type strains across six generations (see Fig. 1).

**Fig. 2:**
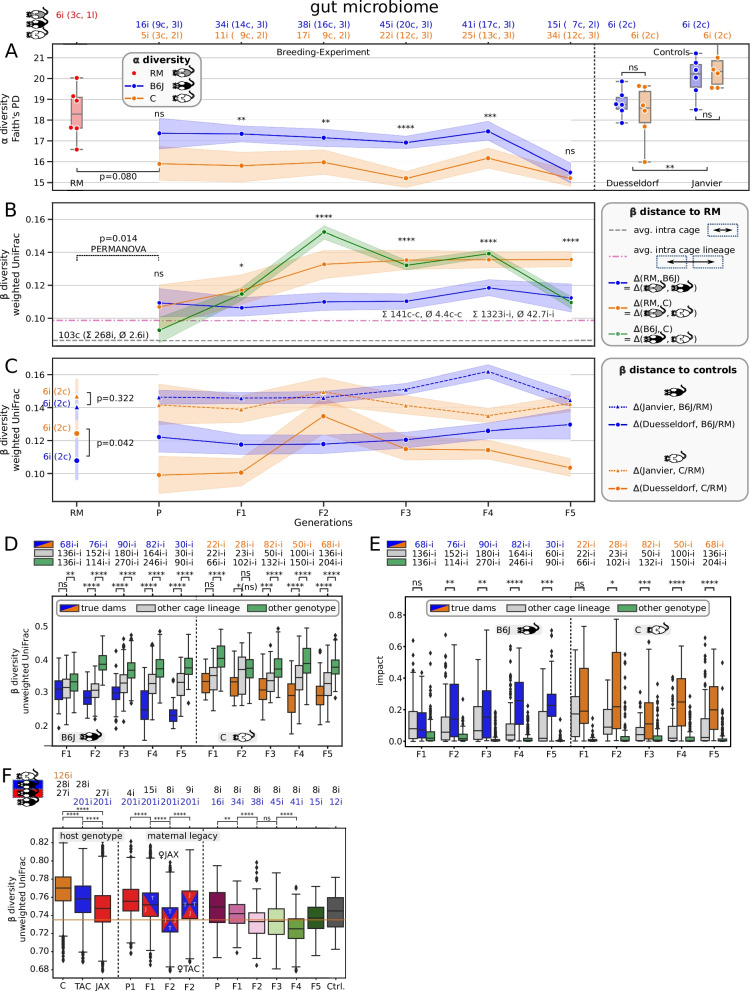
Trajectory of host genotype gut microbiome differentiation. **A** *Y*-axis is Faith’s phylogenetic diversity. *X*-axis is mouse generation or control group. Labels on top list numbers of individual mice (i), cages (c), and cage lineages (l) of which samples were aggregated by color: RM, B6J, and C in red, blue, and orange, respectively. **B** Distances between 6 RM and 303 breeding experiment samples, grouped by host genotype (B6J = blue, C = orange) in terms of weighted UniFrac beta diversity. Green band indicates distance between host genotypes, not to RM. Gray dashed line is the mean pairwise distance between individuals housed in the same cage, i.e., between biological replicates; 103 cages with 268 individuals and 2.6 individuals per cage on average were considered. Magenta dashed line is the mean distance of individuals from two different cages of the same host genotype, same cage lineage and same generation; 1323 pairs of individuals (i-i) were considered with 42.7 i-i pairs on average per cage lineage and generation; considering housing, there are 141 different cage-to-cage (c-c) pairs with 4.4 c–c pairs on average per cage lineage and generation. **C** Distances between 24 control and 303 breeding experiment plus six RM samples in terms of weighted UniFrac. We grouped control samples into host genotype and Janvier vs. Duesseldorf, such that each group consisted of six mice housed in two cages. **D** Comparison of similarities between true dam to offspring (= true dams), dam to mice of same generation, other cage lineage (= other cage lineage), and dam to same generation different host genotype (= other genotype) in terms of unweighted UniFrac. Top label indicates the number of pairwise distances. **E** Impact of maternal microbiome on offspring microbial composition in terms of source tracking for true dam to offspring (= true dams) and dam to mice of same generation, other cage lineage (= other cage lineage), and other host genotype (= other genotype). **F** Joint analysis with Robertson et al. data. The *y*-axis is unweighted UniFrac. First three boxes summarize pairwise distances between our C and Robertson’s mice, our B6J and Robertson’s TAC, and our B6J and Robertson’s JAX mice in orange, blue, and red, respectively. Next four boxes relate our B6J mice with Robertson’s P1, F1, and F2 generation, where the latter is split into maternal JAX and maternal TAC mice. Subsetting Robertson’s F2 maternal JAX mice, the last seven boxes summarize distances to our six generations and B6J controls. We used Mann-Whitney-Wilcoxon for all statistical tests with Benjamini-Hochberg correction for multiple testing (ns: not significant, ****p ≤ 0.0001, ***p ≤ 0.001, **p ≤ 0.01, *p ≤ 0.05)

**Fig. 3:**
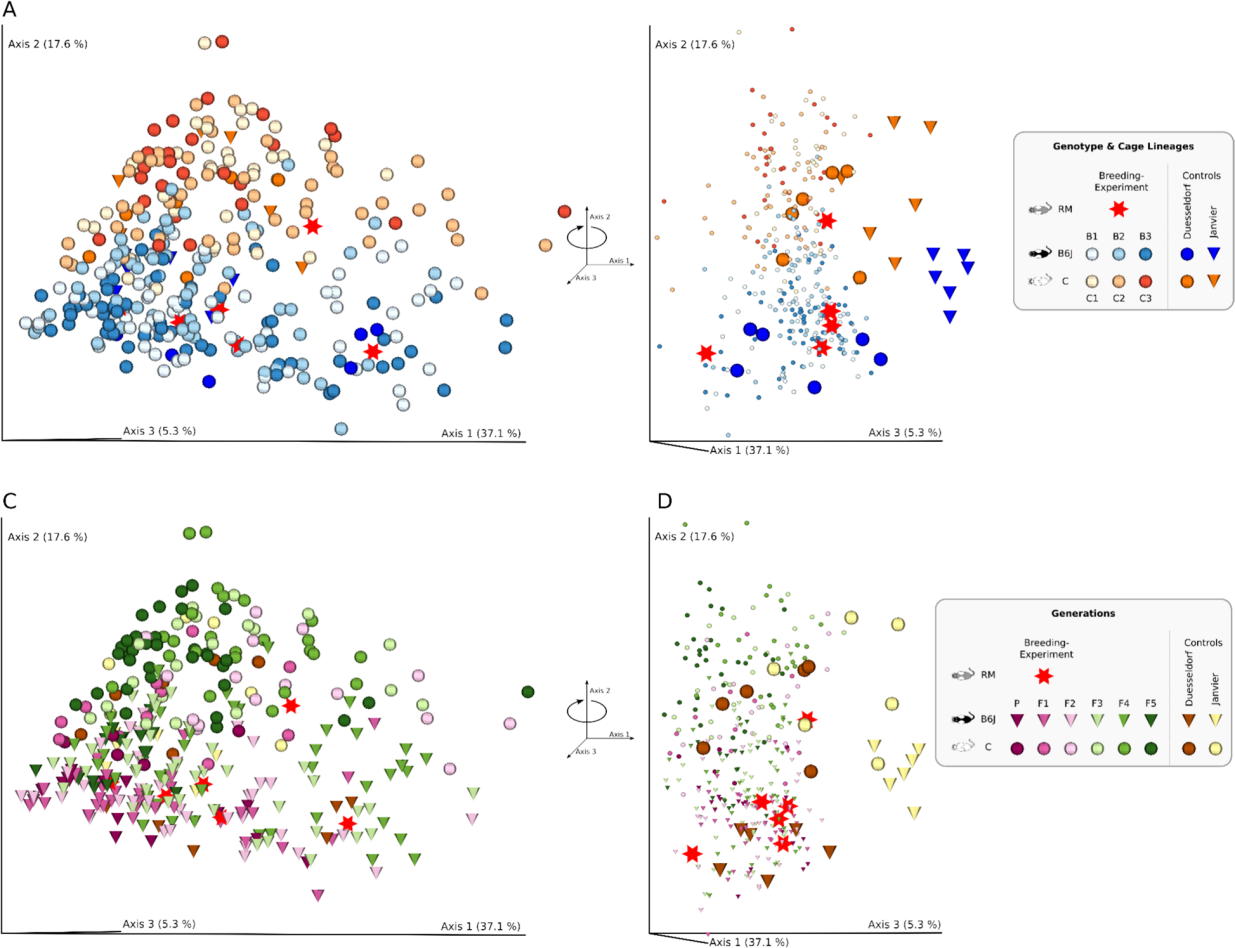
Gut microbial diversity. PCoA of weighted UniFrac distances for 333 colon samples. **A** Colored by host genotype and cage lineage. **B** Rotation of A along Axis 2. **C** Same PCoA as in A, but color here indicates generation. **D** Rotation of C along Axis 2

Our microbial gut data show a robust overriding impact of host genotype over maternal legacy for constant environments. Interestingly, this effect is not yet pronounced in the P generation (*p* = 0.08, two-sided Mann-Whitney-Wilcoxon test) but in all that follow (Fig. 2A: *p* < 0.01, except F5 and Fig. 2B: *p* < 0.03, except *p* = 0.91 for P). The unproportionally smaller number of B6J mice in generation F5 (10.9 mice on average but only 0, 9, and 6 mice in F5 for cage lines B1, B2, and B3, respectively) led to a significantly smaller sampled microbial diversity (Figure S1A: *p* < 0.014 for B2, *p* < 0.004 for B3) and is primarily prohibiting detection of the above mentioned effect. Displaying the data as a PCoA of weighted UniFrac distances reveals two distinct clusters belonging to each of the host genotypes respectively (Fig. 3). Although considered wild types, both host genotypes have different immune system responses, which probably drives the differential microbial composition in the gut, as the main host-microbiome interface in mammals. The significantly lower alpha diversity of C animals persists even when correcting for the number of founding sires in the P generation: three vs. one for B6J and C, respectively (Figure S2).

The RM mice are generated by mating female B6J with male C mice. The high microbial similarity between host genotypes at the P generation (no significant difference, see above) seems to favor the maternal legacy effect; the weighted UniFrac distance between host genotypes (green line in Fig. 2B) is lower than the average distance between any pair of mice from different cages within the same cage lineage (this comprises host genotype, magenta dashed line). In accordance, the RM microbiome is more similar to B6J Duesseldorf controls than to C Duesseldorf controls (Fig. 2C, *p* = 0.042, two-sided Mann-Whitney-Wilcoxon test). Due to open instead of individually ventilated cages, control mice were exposed to a more relaxed environment and might therefore lack significant differences in alpha diversity (right part of Fig. 2A: *p* = 0.80 for Duesseldorf and Janvier controls, two-sided Mann-Whitney-Wilcoxon test). However, controls show strong separation by host genotype in beta diversity (*p* < 0.007 for all four tested metrics, PERMANOVA test with 9999 permutations). The microbiome between RM and mice of the P generation is significantly different (PERMANOVA tests with 9999 permutations on weighted UniFrac: *p* < 0.016), which might result from the relatively invasive embryo transplantation with preceding skin disinfection as an environmental distortion. This would explain the drop in alpha diversity, although not being significant (*p* = 0.080, two-sided Mann-Whitney-Wilcoxon test). To explain the significant microbial dissociation in all following generations (F1 to F5) though, we must favor host genotype over maternal legacy, especially since microbiomes started quite homogeneously in the P generation but drifted apart from F1 onwards, while we kept the environment constant. The relatively constant trajectory (blue line = B6J in Fig. 2B) and the continuously increasing difference (orange line = C in Fig. 2B) emphasize that the microbiome of the hybrid RM mice is dominantly that of B6J mice, and that there must be an active shaping in the C animals.

Despite the clear dominance of host genotype, environmental aspects easily exceed this effect, as can be seen by the significantly higher alpha diversity (Fig. 2A, *p* = 0.0013) of Janvier control mice, which were bought from a commercial vendor and only acclimatized for 1 week in our local facility prior to sampling at 15 weeks of age and the generally larger beta-diversity distance of mice from our breeding experiment with Janvier controls, compared to Duesseldorf controls (Fig. 2C: dashed vs. solid lines, respectively). The environment is probably furthermore a limiting factor for the degree of host genotype-specific tailoring of the microbiome. Our narrow environment (autoclaved cages, autoclaved rodent chow and autoclaved bedding, acidified water, individually ventilated cages) provided a restricted set of microbes the hosts could source from, such that host genotype differences peak around F2 and probably show intergenerational cycling thereafter [65, 69]. This would explain the flips in distance of C to control mice (orange lines in Fig. 2C), whereas distances of B6J to control mice remain relatively stable as only the C host genotype actively tailors its microbiome away from the shared starting point, which is already B6J like, in the P generation.

### Exploring family relations confirms the presence of a maternal legacy effect

The observation of C mice’s microbiome distance to RM increasing faster and stronger than between B6J mice and RM is a result of a maternal legacy effect as the mothers of the RM mice were of the B6J host genotype. In fact, RM samples are closer to B6J than to C Duesseldorf controls (*p* = 0.042, two-sided Mann-Whitney-Wilcoxon test, Fig. 2C).

As we established three cage lineages per host genotype, we could investigate maternal legacy in detail by comparing microbial distances within true family relations, i.e., individuals with their *true dams*, and nonfamily relations, i.e., distances to dams of *other cage lineages* but the same host genotype and last, distance to dams from the *other host genotype* (Fig. 2D). Except for the F1 to F2 relation in C, we observe significant differences between the three categories with *true dams* showing the smallest distances towards their children. Using the SourceTracker [70] tool to estimate seeding capacity of alternative microbial sources to compose the offspring’s microbiome confirms that the *true dams* microbiome has the strongest impact (Fig. 2E).

The maternal legacy effect might explain the dip in alpha diversity of B6J mice in generation F5 as well. Plotting the diversity by cage lineage (Figure S1) shows that samples in generation F4 of cage lineage B1 (blue) have exceptionally high alpha diversity, compared to B2 (orange) or B3 (green), quantified as Faith’s PD or as number of observed features. Although B3 significantly loses alpha diversity in F5, the mean across cage lineages in F5 also unproportionally suffers from a lack of B1 samples, with presumably high(er) diversity. This indicates that diversity can be crucially impacted by maternal legacy per generation.

**Fig. 4:**
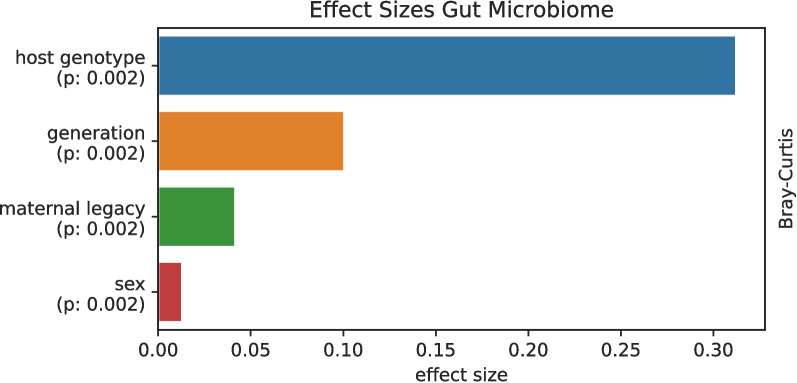
Effect sizes analysis. Forward step redundancy analysis with a linear model composed of host genotype, generation, sex, and cage lineage (= maternal legacy) on Bray-Curtis distances

Effect size analysis on gut data confirms dominance of host genotype. Mouse generation and maternal legacy have smaller but significant effect sizes, which has been reported previously, pointing out the importance of the mother in murine microbiome experiments (Fig. 4).

### Joint analysis with independent data corroborates effects of host genotype and maternal legacy

In [65], authors investigated whether cohousing or F2 littermates, which we consider as the amalgamated effect of host genotype and maternal legacy, would lead to a more homogeneous microbiome prior to performing murine studies. Obtaining mice of very close host genotypes, namely the substrains C57BL6/J and C57BL6/N from two different vendors Jackson Laboratories (JAX) and Taconic Farms (TAC), respectively, they concluded that F2 littermates had a significantly higher impact on microbial standardization than cohousing. Thanks to published raw sequences from colon samples and prompt support with metadata (personal communication), we were able to perform a joint analysis.

We quantified the microbial distances between our C host genotype (*n* = 126 mice of generations P to F5 and controls) and Robertson’s parental and littermate mice of both vendors (*n* = 28 TAC + *n* = 27 JAX), which are of a B6-like host genotype, as the orange box in Fig. 2F. These distances are significantly larger than distances between our closely matching B6J (*n* = 201 mice, *p*≪ 10^−4^) host genotype and Robertson’s TAC (*n* = 28, blue box, *p* ≪ 10^−4^) or JAX (*n* = 27, red box, *p*≪ 10^−4^) mice. This emphasizes the strong impact of host genotype on microbial composition. The decrease in distances of host genotype matching mice between different vendor-dependent sub-strains (blue to red) aligns to the fact that our mice originate from a Jackson Laboratories purchase. This difference is significant and interestingly of similar magnitude as mismatching host genotypes (orange to blue). Practically, this could imply that not only the genotype of the utilized mice but also the substrain must be normalized for future murine microbiome experiments.

The maternal legacy effect probably complicates matters. Robertson et al. generated F2 hybrids of vendor sub-strains in two fashions: ♀JAX used JAX dams and TAC sires, while ♀TAC used TAC dams and JAX sires. Comparing distances between our *n* = 201 B6J mice and Robertson’s *n* = 8 F2 ♀JAX samples (sixth box in Fig. 2F) or Robertson’s *n* = 9 F2 TAC samples (seventh box in Fig. 2F) illustrates that maternal legacy shapes significantly different microbiomes (*p* ≪ 10^−4^). As we employed a pure B6J host genotype of dam and sire for breeding, it is convincing that distances to Robertson’s F2 ♀JAX samples are significantly smaller than to F2 ♀TAC. This finding lets us specify the above recommendation to normalize or at least record female lineage for murine microbiome experiments.

Significantly decreasing distances from P1 (fourth box) to F1 (fifth box) and F1 to F2 (sixth box) recapitulates Robertson’s recommendation to generate F2 mice prior to experimentation. Furthermore, stratifying Robertson’s 27 JAX mice (but not the 28 TAC mice) by generations (fourth to sixth box in Fig. 2F) shows that they indeed become significantly more similar to our *n* = 201 B6J mice over time — in accordance with our previous observations. Despite marked biological and technical differences between Robertson’s and our microbiome profiling, it is interesting to see that our B6J samples become significantly more similar (except F3 and F5) to Robertson’s F2 JAX samples with preceding generations (seven rightmost boxes in Fig. 2F). This might point to a universal host genotype-specific core microbiome and warrants further investigation. Due to different variable 16S rRNA gene regions, we assume incompatible taxonomic assignments (cf. tremendous shifts in Bacteroidota/Firmicutes_A ratio in Figure S3) and therefore refrain from further investigations on taxonomic features.

Taken together, we concur with Robertson et al. that F2 littermates should become the gold standard for microbial studies, and we add that host genotype down to a level of substrain together with maternal legacy must be controlled for.

### Skin microbiome shows effects of “host genetics” but lacks maternal legacy

We sampled the skin of the left earlobe of all mice in addition to the previously discussed colon samples by processing the whole tissue in order to also capture sub-epidermis bacteria, e.g., in hair follicles [71]. Lower biomass led to fewer reads, and, subsequently, more samples were lost through quality control, invalidating application of statistical tests due to low sample numbers for some of the following comparisons.

**Fig. 5:**
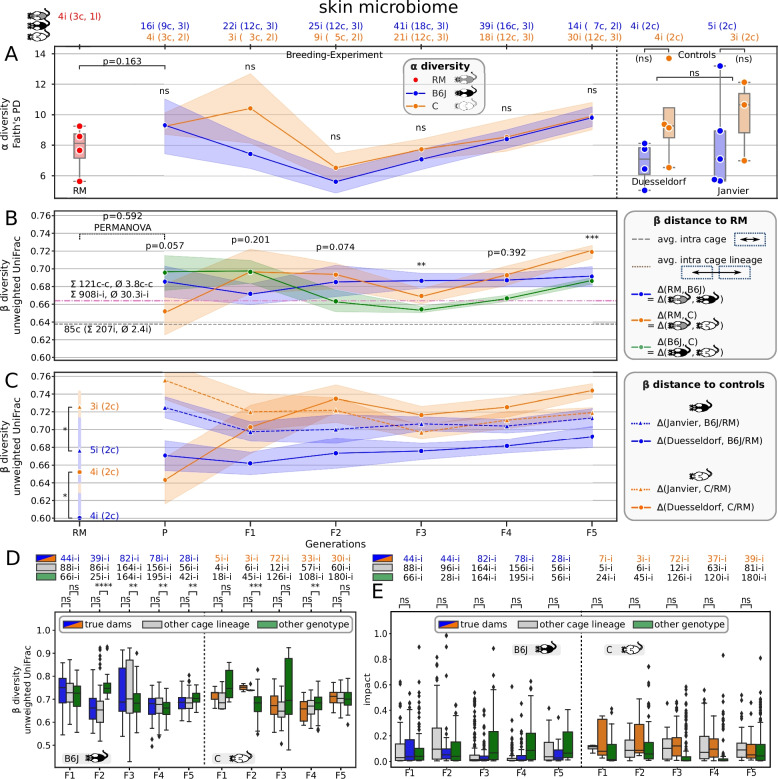
Trajectory of host genotype *skin* microbiome differentiation. Structure of this figure is identical to Fig. 2 but for skin microbiome samples. Please consult the legend of Fig. 2 for details. Differences are as follows: **F** is missing, since Robertson et al. did not collect skin samples. **B** and **C** show unweighted instead of weighted UniFrac distances

As in the gut, host genotype shapes the skin microbial communities in a genotype-dependent manner (Figure S4), although the results are not as decisive (Fig. 5B: two-sided Mann-Whitney-Wilcoxon tests: generation P (*p* = 0.057), F1 (*p* = 0.201), F2 (*p* = 0.074), F3 (*p* = 0.007), F4 (*p* = 0.392), F5 (*p* < 0.001)). The RM skin microbiome flips between being more similar to B6J and C three times throughout the P to F5 generations. Using control mice as reference instead (Fig. 5C), the skin microbiome seems to be more similar to B6J for all but the P generation. Interestingly, alpha diversity (measured as Faith’s PD, Fig. 5A) is never significantly different across any generation; this is also true for the alternative metrics “Shannon diversity,” “Chao1,” and “observed features,” i.e., the raw number of different ASVs.

We were not able to measure a maternal legacy effect in the skin samples, neither by comparing alpha diversity (Figure S5), beta diversity (Fig. 5D), nor by source tracking (Fig. 5E). Again, low sample numbers prohibit statistical testing, but microbial alpha diversity between Janvier and Duesseldorf controls seem to be markedly different (Fig. 5A), pointing to a stronger “environmental” impact on microbial composition.

Due to the cohousing of mice and their social nature, we cannot rule out the possibility of microbes from the gut transferring to the skin through factors like coprophagy and mutual grooming (cf. Figure S6 for gut/skin differences). Using five chow and five bedding control samples of the lots used for mice housing in addition to gut microbiome samples, stratified by host genotype and generation as “sources,” we quantified the contribution of community assembly in the skin (“sink”) via source tracking (Figure S7). Note that different read coverage between sinks and sources likely skews results. The source tracking analysis shows that the skin microbial community is only composed of 6% on average of microbes found in host genotype matching gut samples of the same generation. Microbes from the “opposite” host genotype gut microbiome account for negligible 1% on average. Cage bedding material (5%) and mice chow (16%) had similar or approx. threefold stronger impact on skin microbiome assembly, whereas the huge majority of community composition remains unknown (68%), which might actually represent the “true” skin microbiome. We conclude that environmental effects dominate the skin microbiome with clear imprinting of host genotype tailoring but no detectable maternal legacy effects.

### Select taxa like *A*. *muciniphila* are linked to host genotype in both gut and skin microbiomes

**Fig. 6:**
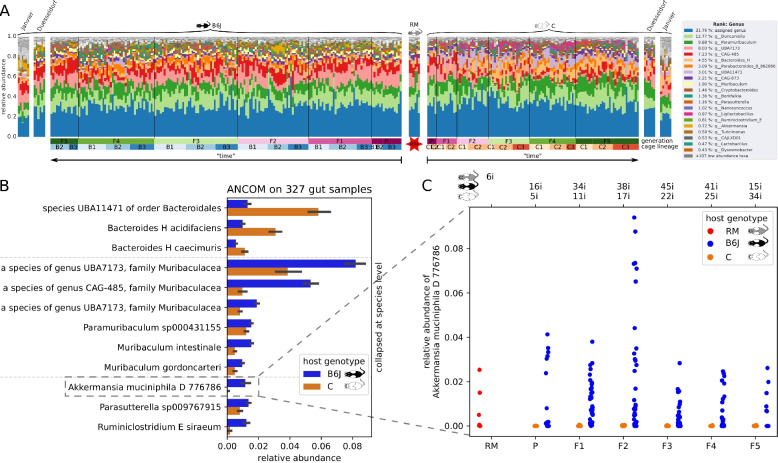
Gut taxonomy. **A** Taxonomic composition of 333 gut microbiome samples on genus level. Purple to green bar indicates generations, and blue and orange bars indicate cage lineage. **B** Mean relative abundance of significantly differentially abundant species between host genotypes, determined via ANCOM. Further, 21 species were excluded due to very low abundances. **C** All relative abundances of species *A. muciniphila* D 776786, stratified by generation (*x*-axis) and host genotype (hue)

Bacteria of the phyla Bacteroidota (75.13%) and Firmicutes_A (17.73%) dominate the baseline gut microbiota, whereas the Proteobacteria (2.28%) play a subordinate role (Figure S8A). In contrast, the phyla Firmicutes_D (45.88%) and Proteobacteria (20.53%) make up the majority of skin microbiota, whereas the Bacteroidota (12.32%) were much less abundant (Figure S8B). The taxonomic composition at genus level is presented in Fig. 6A and Figure S8C for the gut and skin, respectively. Both host genotypes shared most of the taxa in both gut and skin microbiomes over the generations and cage lineages. However, singular genera occurred preferentially only in B6J or C in the skin, as well as in combinations of generations or cage lineages in both the gut and skin (Figures S5 and S6). Collapsing the 946 gut ASVs to 102 named species level, ANCOM found 33 species to be significantly differentially abundant between B6J and C host genotypes, of which 21 had very low abundances (Fig. 6B). Higher abundance of three species of the *Bacteroides* genus in C mice and higher abundance of six species of the Muribaculacea family, *Parasutterella*, *Ruminiclostridium*, *Eubacterium siraeum*, and *A. muciniphila* in B6J mice, suggest a host genotype-specific enrichment of particular taxa. Stratifying the relative abundance of *A. muciniphila* per generation (Fig. 6C) shows an equally high abundance in the RM foster mothers, corroborating that the host genotype-dependent shaping of the gut microbiome works through modulation of individual taxa transferred by the mother via maternal legacy.

### Blood serum metabolites correlate with host genotype and its colonizing gut microbiome

**Fig. 7:**
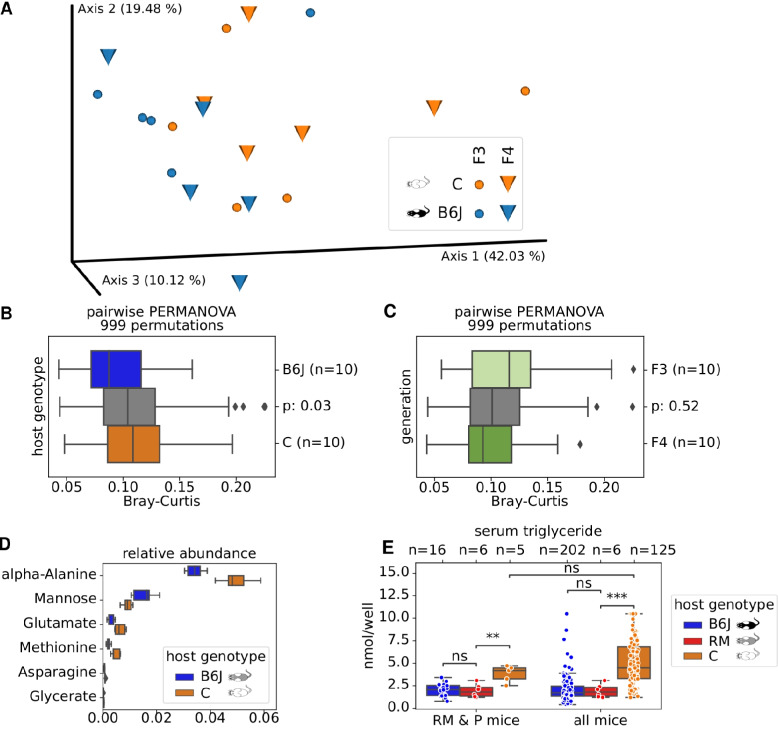
Gut metabolite diversity. **A** PCoA of Bray-Curtis distances for 40 blood serum samples, obtained from 5 mice each in F3 and F4 of cage lineages B2, B3 and C3, and C2, respectively. We quantified 41 metabolites per sample. **B** Pairwise Bray-Curtis distances within host genotypes (B6J = blue, C = orange boxes) and between (gray) are significantly different as assessed via PERMANOVA. **C** Pairwise Bray-Curtis distances within “generations” (F3 = light green, F4 = dark green) and between (gray) are not significantly different as assessed via PERMANOVA. **D** Relative abundances of six metabolites found to be significantly different by dsFDR between host genotypes. **E** Serum triglyceride concentrations, stratified by host genotype (hue) for only early generations (= RM & P mice) and all generations (= all mice). Significance was assessed by Mann-Whitney-Wilcoxon with Benjamini-Hochberg correction

We quantified serum triglycerides in all 333 mice, to further investigate host and microbiome interaction. Interestingly, we found the same host genotype-dependent correlation as with the gut microbiome, namely very similar triglycerides levels (*p* ~ 0.96, two-sided Mann-Whitney-Wilcoxon test) between RM and B6J mice (Fig. 7E), and significantly different (*p* < 0.0005) levels between RM and C mice. However, as triglyceride serum levels are already significantly different (*p* < 0.009) between RM and C in the first generation (P) after embryo transfer, a generation for which we could not detect microbial differences, we conclude that triglyceride levels are directly controlled by host genotype which in turn might help in shaping the microbiome.

It has been previously reported that the blood serum is a means to communicate gut microbial differences into the host organism. We therefore measured 41 serum metabolites via GC–MS of 10 selected mice of B6J and C host genotypes each (Fig. 7A). To capture temporal changes, we sampled mice of the generations F3 and F4 for which we found pronounced microbial differences. To exclude maternal legacy, we intentionally sampled different cage lineages in both generations, i.e., B2, C3 and B3, and C2 in F3 and F4, respectively. Quantified as Bray-Curtis pairwise distances, we found significant (*p* = 0.02, PERMANOVA with 999 permutations) differences in serum metabolite profiles between host genotypes (Fig. 7B). Closer inspection via dsFDR found 6 out of the 41 metabolites to be differentially abundant between host genotypes (Fig. 7D). From our limited metabolome data, we can only speculate about directionality, but the observed differences might be a direct result of gut microbial metabolite production, which penetrates into the host’s bloodstream. We could not detect differences (*p* = 0.52, PERMANOVA test with 999 permutations) between generations F3 and F4 (Fig. 7C).

## Discussion

### The host genotype shapes its host’s microbiome

To what extent the host genotype affects the microbiome composition, and whether this effect is general or impacts only certain taxa, is still subject to debate. The littermates are regarded as gold standards in microbiome standardization of experimental groups [65]. The immune system dwells with the microbial world, and extreme immune-altered host genotypes clearly influence the composition and diversity of the gut microbiome [16–18, 20]. Nevertheless, studying the impact of unmodified host genotypes on the microbiome is more difficult, because the effects are usually softer and cannot be directly attributed to particular engineered genes. However, the contribution of particular genes or genomic quantitative trait loci (QTL) to the microbiome tailoring [40, 72] or associations to microbial taxonomies and especially to particular genera such as *Bifidobacterium* has been demonstrated [73, 74]. The host genotype possibly acts on the microbiome by the innate and adaptive immune systems, which sequentially shape the gut microbiota, lipid metabolism, and stat3 phosphorylation [75], and thus applies different evolutionary within-host selection forces to the microbial communities [76]. The two mouse strains B6J and C differ substantially in their immune responses to various infectious agents and are seen as prototypes for Th1 and Th2 immune response, respectively [77, 78], which may induce through microbiome-immune system interaction, different microbial communities [79].

Our approach studies whether differences in the microbiome can occur over generations in littermates of different host genotypes (B6J and C) in a constant environment, after a natural course of colonization with a common microbiome of B6CF1-recipient mothers. Moreover, the two main microbiome ecological niches, the gut and the skin, are considered, since knowledge on body sites other than gut is currently sparse.

We demonstrated that the host genotype essentially contributes to the active shaping of the gut microbiome and has a powerful influence on the host’s metagenome. This influence is exerted both directly through its stable genome, and in addition indirectly through tailoring of the flexibel composition of microbes that colonize the host. Despite the limitations in the profiling of the skin microbiome mentioned below, it seems that role of the host genotype is less decisive for the formation of the skin microbiome, which might be rather environmental dependent. Since the host genotype is heritable, this is an important factor in the microbiome evolution over the generations in spite of possible changes in the environment [80, 81].

Interestingly, we observed that the microbiome of our B6J mice was more similar to Robertson’s B6J than to Robertson’s B6N mice despite marked spatiotemporal (2019 vs. 2022, Canada vs. Germany), biological (sampling at 15 vs. 8 weeks of age, acidified vs. nonacidified water, cages changed weekly vs. bimonthly, commercial pelleted food vs. autoclaved chow), and technical (different technicians, V34 vs. V4 16S rRNA gene region, different sequencing centers) differences. This finding sustains that host genotype differences at substrain level (B6J vs. B6N) are still enough to produce host genotype-related shaping of the gut microbiome and might point to a universal host genotype-specific core microbiome, which warrants further investigation. Despite the clear dominance of host genotype, environmental aspects easily affect the microbiome tailoring, as can be seen by the significantly higher alpha diversity of Janvier control mice compared to Duesseldorf mice (Fig. 3A). The environment is probably a limiting factor for the degree of host genotype-specific tailoring of the microbiome in our experiment. Thus, the environment and host genotype decisively influence the composition of gut murine microbiota [33].

Whether or not the host genotype differences in the skin microbiome of the earlobe are actively tailored by the mice or is an indirect reflection of the active tailoring of the gut microbiome with subsequent spreading of these microbes to their skin cannot be unambiguously answered from our data. Fewer viable bacteria than predicted by bacterial DNA profiles colonize the skin surface [71]. It is plausible that bacterial environmental noise might impact the recording of the skin microbiome. Nevertheless, the environmental noise should partially originate from the viable skin-associated bacteria that are predominantly located in hair follicles and other cutaneous invaginations [71] and correlate taxonomically. Our analysis included the hair follicle and invaginations by including the whole ear lobe skin and not only bacteria from the upper layers of the epidermis. Moreover, since mammalian skin is a highly specialized habitat, capable of strong selection from available species pools [82], filtering thus probably occurs by host own forces and shapes the pool of bacteria that lead to this type of contamination. The influence of environmental noise on the microbiome could be partially reduced by the RNA-based profiling as a preferred screening method [82]. Nevertheless, even a RNA-based profiling still records the living contaminants such as the ones acquired by coprophagy or from cage environmental sources such as bedding/food. Although we used a DNA-based profiling, the source tracking analysis shows that the impact to which the environmental contaminants drive microbial composition of the skin samples plays only a subordinate role (Figure S7), implying a host genotype active tailoring also in the case of skin microbiome but to a much lower extent as for the gut. The interactions between the host immune system and skin are presumed to be much less intensive. External skin is in general more prone to environmental conditions and thus much harder to control for.

### The host genotype also shapes its metabolome

Multiple health and disease markers are correlated with the composition of the gut microbiome in humans [83]. In addition, the human gut microbiome affects the host serum metabolome and is linked to insulin resistance [84].

Using GC-MS-based metabolomics, we demonstrated differential expression and abundances of serum metabolites among selected B6J and C mice. Our findings indicate that the differences in microbiome could modulate together with the host genotype the expression of systemic markers (see Fig. 7).

### The host genotype enriches specific microbial taxa

Analysis of the taxa variation between B6J and C mice in our study revealed that particular taxa were enriched by host genotypes. Interestingly, [85] observed 22 taxa to have a significantly higher abundance in B6J than C mice, including *Akkermansia* and *Ruminococcus*. (We assume equivalence between genera *Ruminiclostridium* and *Ruminococcus* in our data, as 99.6% of *Ruminiclostridium* reads were classified as *Ruminococcus* when using Horne et al. outdated GreenGenes version.) The similar enriched abundances of *Akkermansia* and *Ruminococcus* in both studies, regardless of the experimental design, suggest that the gut environment of B6J but not of C mice is auspicious for these taxa. This may be due to increased availability of niche energy source Muc-2 in B6J, since *A. muciniphila* has the ability to degrade Muc-2 O-glycans in vitro [86]. *A. muciniphila* is an important pathobiont influencing numerous animal experimental phenotypes and accounts for 1–5% of the gut microbial community in healthy human adults, being a marker of a healthy microbiome and increasing the integrity of the intestinal barrier in both humans and mice [87]. There are obvious relationships between *A. muciniphila* and chronic inflammatory metabolic diseases such as type 2 diabetes, obesity, and IBD [88–90]. Interestingly, *A. muciniphila* accounted for up to 9% of the gut microbiota of the B6J but not of the C mice of our study (Fig. 6C).

Overall, most of the microbial genera were shared by both host genotypes and inherited overall generations (Fig. 6A), although differential taxa abundances occurred between host genotypes (Fig. 6B), whereas singular genera were present only in some host genotypes, cage lineages, and generations. Moreover, particular genera jumped over some generations, probably under the detection limit, and reappeared in a later generation (Figures S9 and S10).

### The maternal legacy imprints the microbiome

The intergenerational changes recorded in our data are in accordance with previous studies. Minor changes of intestinal microbial composition and/or function across generations were previously reported in [69] and [65], when inbred mouse strains were transferred into new facilities. Moreover, such studies suggest that even the more resilient wilding’s gut microbiota [15] are expected to change as animals are housed under laboratory conditions [91]. An expected host genotype independent finding was thus the cage lineage specificity, emphasizing the role of maternal legacy in microbiome heredity (Figs. 2A, 3D and E) similar with previous studies [92]. Importantly, maternal legacy does not necessarily mean maternal microbiome if the male remains in the female cage during pup rearing favoring also the paternal horizontal transmission of microbial taxa [65]. Overall, we here documented by microbiome source tracking that the maternal legacy and the dam itself are responsible for most of the gut microbiome transmission to the offspring (Figs. 2E and 4E). In our study, maternal legacy represents the second most important endogenous factor contributing to the shaping of the gut microbiome after host genotype effects.

### The host genotype is dominating maternal legacy, which both shape the host’s microbiome

Multiple studies attribute the host genotype a certain degree of influence on the gut microbiome [29, 30, 37], whereas others attribute to the host genotype a secondary role [34, 36] or no importance in microbiome shaping [93] at all. The authors of [38] examined the host genotype and microbiome data from 1046 healthy human individuals with several distinct ancestral origins who share a relatively common environment and found that the gut microbiome is not significantly associated with genetic ancestry, concluding that host genotype has a minor role in shaping the microbiome composition. Nevertheless, a narrow standardization of human individuals to a level similar to mice studies concerning host genotype (inbreeding) and environmental conditions is not achievable.

Previous work to disentangle the impact of host genotype and maternal legacy on the composition of offspring microbiome only sampled the first offspring generation [33, 34, 37]. Since no significant microbial difference could be detected after embryo transfer, cross-fostering, or cohousing, exactly as in the microbial profiles in our P generation, authors rightfully concluded that maternal legacy dominates any “host genetics” effects, if present at all. Interestingly, a dominance of the host genotype over the maternal inoculation was also documented by cross-breeding of inbred mice [31]. However, both scenarios were based on a temporally limited observation.

The straight experimental design of our study, spanning seven generations of mice, with identical exogenous parameters regardless of the endogenous host genotype dichotomy, clearly shows that microbial differences manifest in the F1 generation and further increase over time, at least for constant environments. We therefore argue that our data is compatible with previous contradicting findings; however, our longer temporal sampling suggests the opposite conclusion, namely that “host genetics” dominates maternal legacy, which for itself, but to a lesser extent, is also acting in tailoring the microbiome. According to a forward step redundancy analysis on Bray-Curtis dissimilarities, host genotype turned out to be the main driver of gut microbial diversity with an effect size of 0.312, followed by generation (0.100), “maternal legacy” (0.04205), and sex (0.013) (Fig. 4). The weak signal on skin microbiome indicates that the microbiomes of different anatomical sites are driven with different power by intrinsic and extrinsic influences such as host genotype and environment. The remaining charred size effects for the microbiomes were possibly driven by the common environmental factors in this study. It is reasonable at this time point to hypothesize that the gut microbiome as an “intern” microbiome, without strong environmental contact, is either prone to changes by host own factors such as host genotype, whereas microbiomes with high environmental contact such as the skin microbiome appear in this study more environmentally dependent.

### Outlook

The implication of the host genotype in shaping microbial communities of further body sites such as of the genital and respiratory mucosa should be addressed in the future. Future research may also document whether the microbiome tailoring by the host genotype can explain why some mice strains are more suitable for particular experimental models than others. For example, it would be interesting to study whether the host genotype-dependent enrichment of the same particular taxa occurs independently in multiple facilities or whether the *A. muciniphila*-dependent phenotypes could be recapitulated in mice strains that behave refractory to *A. muciniphila* enrichment such as C mice. Overall, the host genotype-related shaping of the gut microbiome points to the existence of a universal host genotype-specific core microbiome in inbreed laboratory mouse strains that warrants further investigation.

## Conclusion

Our results conclude that microbial communities at different body sites are driven by different endogen and exogenous factors. While the host genotype strongly influences the active shaping of the gut microbiome, it appears that the skin microbiome is more prone to environmental conditions. Although the microbial genes clearly outnumber genes directly encoded by the host, we propose here that the host genes, as the stable part of the holobiont, play a leading role expressing phenotypes through its microbiome-shaping capacity, possibly through the establishment of universal host genotype-specific core microbiota.

## Supplementary Information

Additional file 1. Metabolites table. Measures targeted metabolites for 40 blood serum samples

Additional file 2. Analysis repository. We performed all the presented analysis and graph generation through a single jupyter notebook. It lists necessary dependencies for full reproducibility. We have outsourced some of the functions into a public 16s rRNA gene analysis code repository: https://github.com/sjanssen2/ggmap. By default, external system calls are submitted to a Slurm cluster. You can instead run on your local machine by providing the optional argument use_grid=False. Additional conda environments can be conveniently created via "cd recipes && make all-env" after cloning the above repository and changing into its working directory. All files necessary for our analysis, the jupyter notebook and a static HTML version of the jupyter notebook for ease of readability are packaged as one zip compressed file

Additional file 3: Supplemental figures 1 to 10. Figure S1: Individual Alpha Diversity of Gut Samples, Figure S2: Impact of number of founding sires on gut alpha diversity, Figure S3: Taxonomic composition of our gut (n=333, labeled as Qiita study 13422) and Robertson's et al. colon (n=120) samples, Figure S4: Skin Microbial Diversity, Figure S5: Individual Alpha Diversity of Skin samples, Figure S6: Differences in body sites, Figure S7: Impact of External Factors on the Skin Microbiome, Figure S8: Taxonomy Barplots, Figure S9: Distribution of Taxa in the gut, Figure S10: Distribution of Taxa in the Skin.

## Data Availability

The trimmed, demultiplexed fastq sequencing data supporting the conclusions of this article are available in the European Nucleotide Archive, PRJEB70879 https://www.ebi.ac.uk/ena/browser/view/PRJEB70879. The GC-MS raw data supporting the conclusions of this article are included within the article as Additional File 1. The analysis notebook and required files to reproduce all statistics and figures is available as Additional File 2 or from https://github.com/jlab/microbiome_benga_hostgenotype.
