# Supplementary figures and images for: The host genotype actively shapes its microbiome across generations in laboratory mice
Source: Microbiome. 2024 Dec 5;12:256. doi: 10.1186/s40168-024-01954-2 (PMC11619136; doi:10.1186/s40168-024-01954-2)

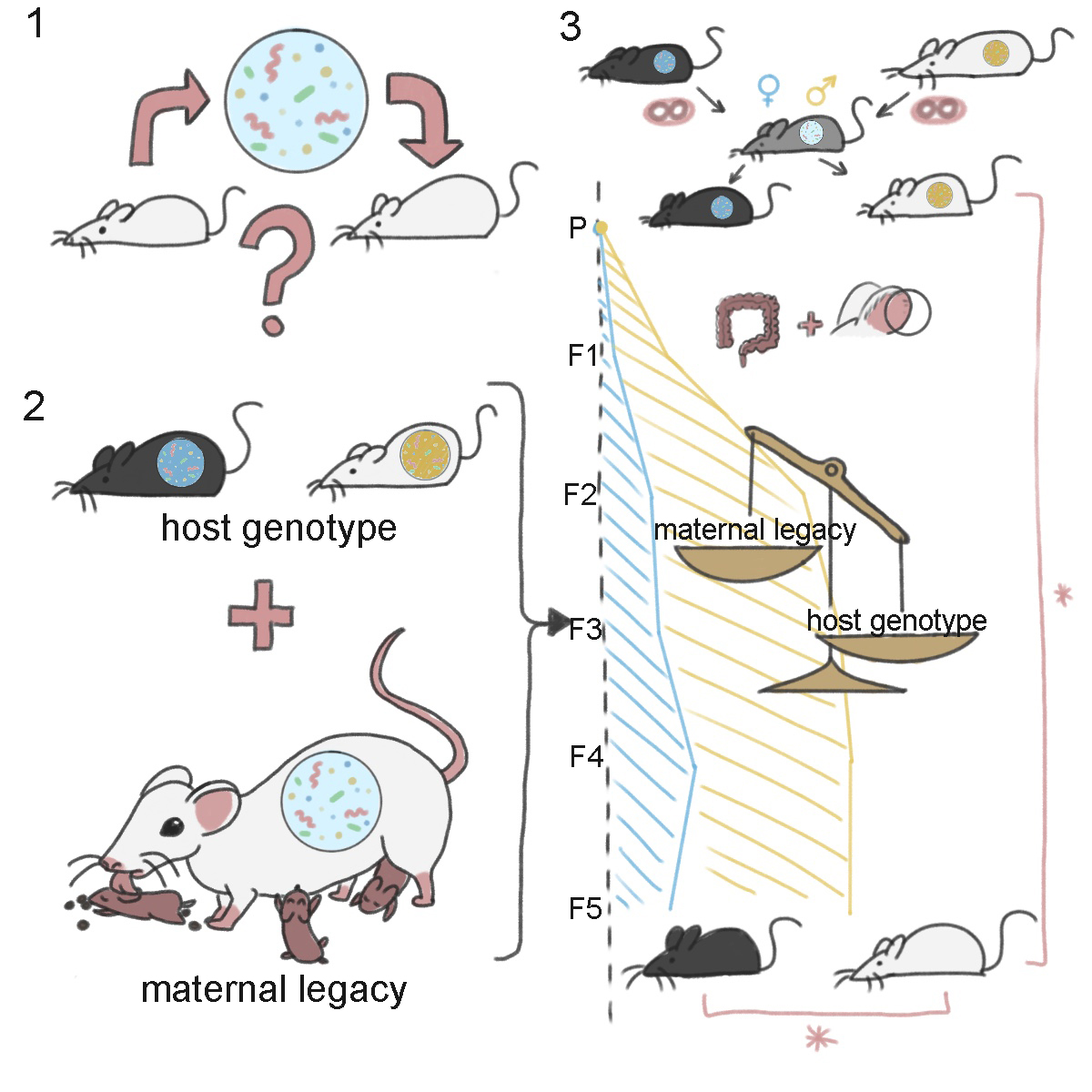

Supplement: Supplementary file 3 — Additional file 2. Analysis repository. We performed all the presented analysis and graph generation through a single jupyter notebook. It lists necessary dependencies for full reproducibility. We have outsourced some of the functions into a public 16s rRNA gene analysis code repository: https://github.com/sjanssen2/ggmap. By default, external system calls are submitted to a Slurm cluster. You can instead run on your local machine by providing the optional argument use_grid=False. Additional conda environments can be conveniently created via "cd recipes && make all-env" after cloning the above repository and changing into its working directory. All files necessary for our analysis, the jupyter notebook and a static HTML version of the jupyter notebook for ease of readability are packaged as one zip compressed file [file 40168_2024_1954_MOESM2_ESM.zip › microbiome_benga_hostgenotype-1.2.0/Figures/GA.jpg]
