# Supplementary material for: The host genotype actively shapes its microbiome across generations in laboratory mice
Source: Microbiome. 2024 Dec 5;12:256. doi: 10.1186/s40168-024-01954-2 (PMC11619136; doi:10.1186/s40168-024-01954-2)
Supplement: Supplementary file 4 — Additional file 3: Supplemental figures 1 to 10. Figure S1: Individual Alpha Diversity of Gut Samples, Figure S2: Impact of number of founding sires on gut alpha diversity, Figure S3: Taxonomic composition of our gut (n=333, labeled as Qiita study 13422) and Robertson's et al. colon (n=120) samples, Figure S4: Skin Microbial Diversity, Figure S5: Individual Alpha Diversity of Skin samples, Figure S6: Differences in body sites, Figure S7: Impact of External Factors on the Skin Microbiome, Figure S8: Taxonomy Barplots, Figure S9: Distribution of Taxa in the gut, Figure S10: Distribution of Taxa in the Skin. [file 40168_2024_1954_MOESM3_ESM.pdf]

Supplement

Figure S1

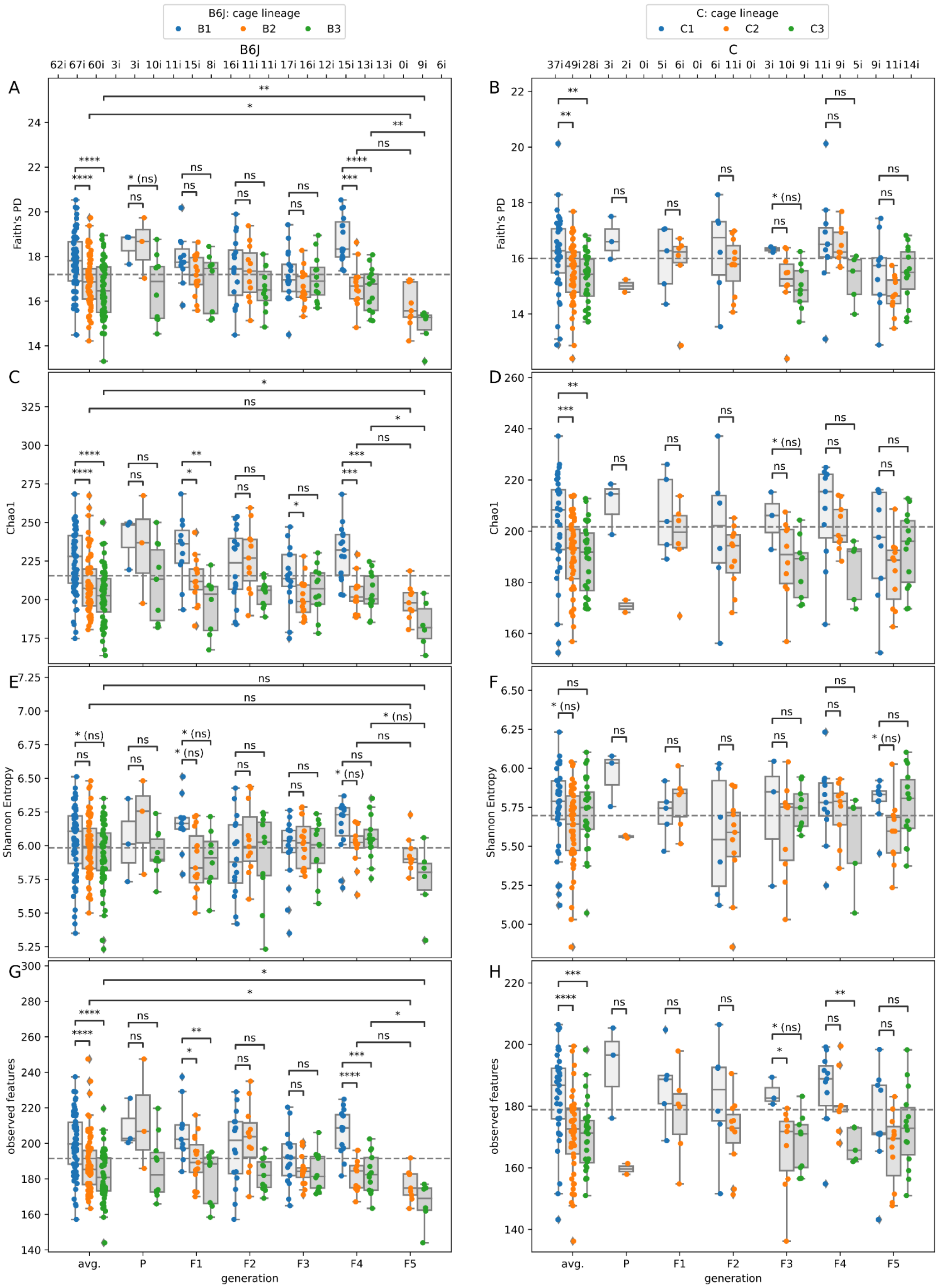

__Figure S1: Individual Alpha Diversity of Gut Samples.__ Left and right columns display alpha diversity of B6J and C samples, respectively. Rows are different alpha diversity metrics: Faith's PD, Chao1, Shannon Entropy and number of observed features, i.e. ASVs. The x-axis stratifies samples into generations, while hue indicates the three different cage lineages. Leftmost x-axis positions, entitled "avg." are samples from all generations lumped

together. We used two-sided Mann-Whitney-Wilcoxon tests with Benjamini-Hochberg correction to assess statistical significance.

Figure S2

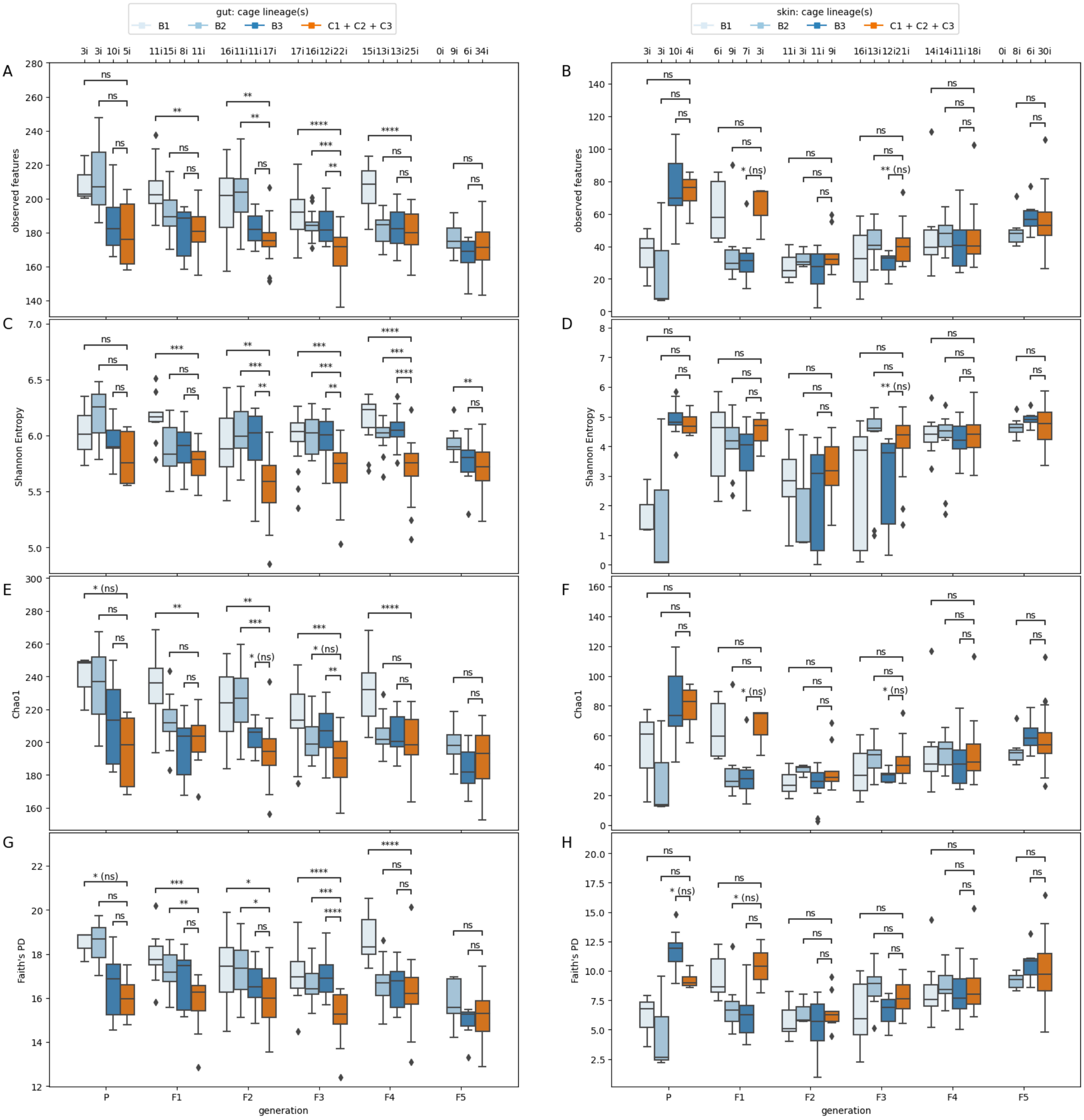

Figure S2: Impact of number of founding sires on gut alpha diversity. *Left and right columns display alpha diversity of gut and skin samples, respectively. Rows are different alpha diversity metrics: Faith's PD, Chao1, Shannon Entropy and number of observed features, i.e. ASVs. The x-axis stratifies samples into generations, while hue indicates samples originating from an individual founding sire in generation P, which coincides with cage lines for B6J and leads to lumping all three cage lines for C into one category. We used two-sided Mann-Whitney-Wilcoxon tests with Benjamini-Hochberg correction to assess statistical significance.*

Figure S3

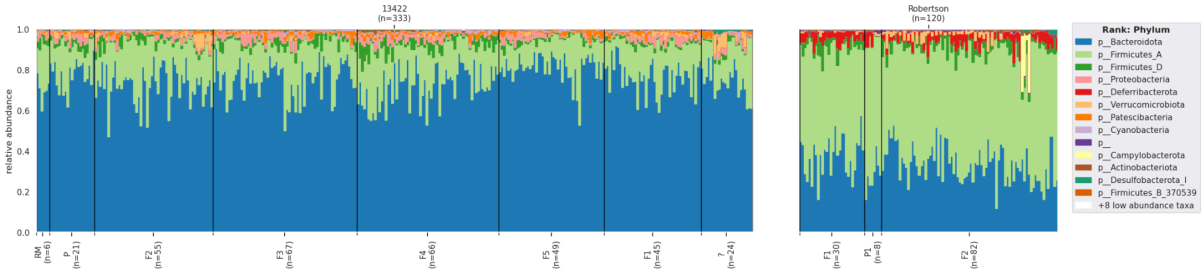

Figure S3: Taxonomic composition of our gut (n=333, labeled as Qiita study 13422) and Robertson's et al. colon (n=120) samples. *Feature counts have not been rarefied. Due to different variable 16S rRNA gene regions, we assume incompatible taxonomic assignments. Exemplary is the difference in the Bacteroidota / Firmicutes_A ratio.*

Figure S4

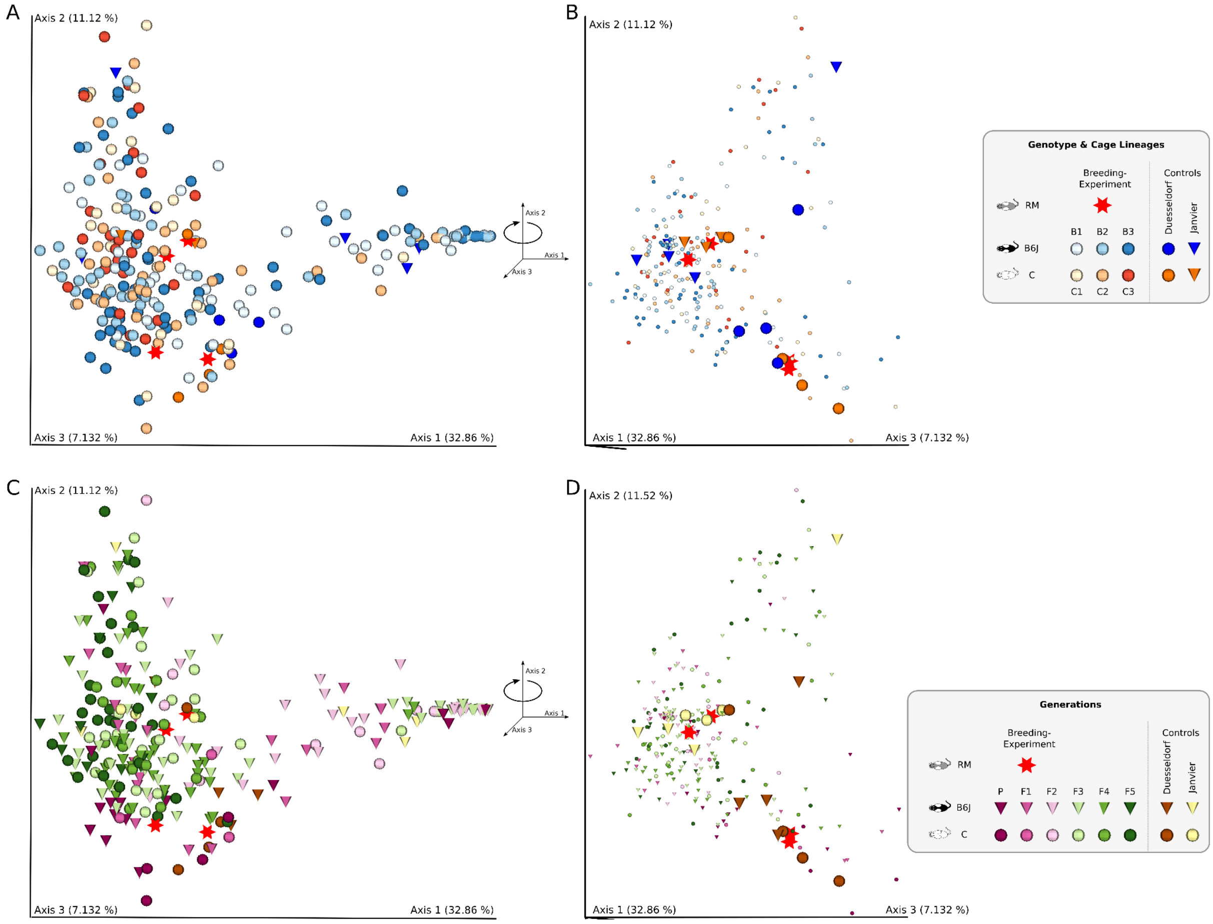

Figure S4: Skin Microbial Diversity. *PCoA of weighted UniFrac distances for 262 external skin of the left earlobe samples. A) Colored by host genotype"host genotype" and cage lineage. Red stars indicate RM samples, while cones stand for Janvier control samples. B) Rotation of Panel A along Axis 2 with decreased icon size of Breeding-Experiment samples to highlight RM and control samples. C) Same PCoA as in Panel A, but color here indicates generation. Spheres indicate C samples; cones indicate B6J samples. D) Rotation of Panel C along Axis 2.*

Figure S5

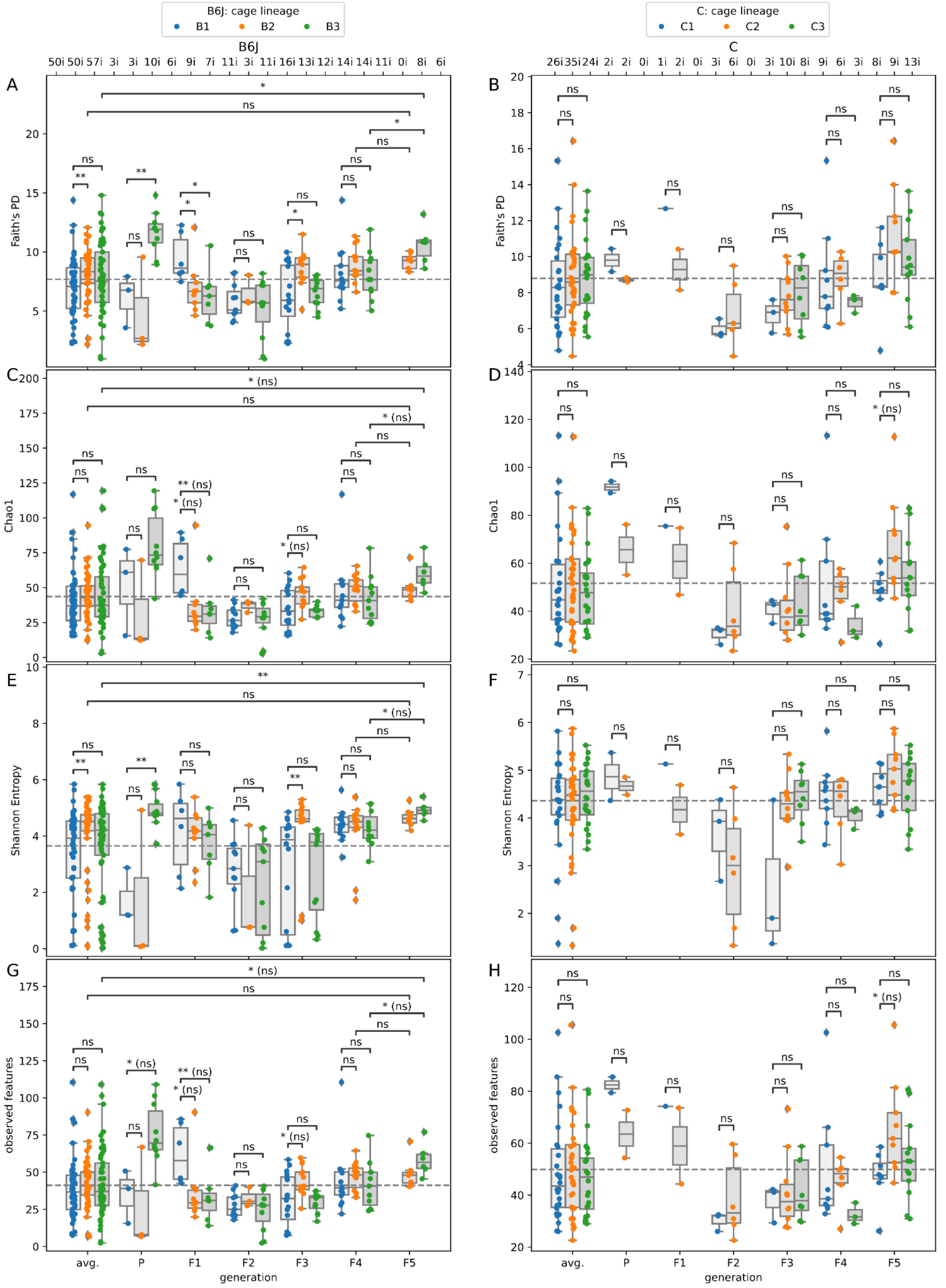

Figure S5: ***Individual Alpha Diversity of Skin samples.*** *Left and right columns display alpha diversity of B6J and C samples, respectively. Rows are different alpha diversity metrics: Faith's PD, Chao1, Shannon Entropy and number of observed features, i.e. ASVs. The x-axis stratifies samples into generations, while hue indicates the three different cage lineages. Leftmost x-axis positions, entitled "avg." are samples from all generations lumped together. We used two-sided Mann-Whitney-Wilcoxon tests with Benjamini-Hochberg correction to assess statistical significance.*

Figure S6

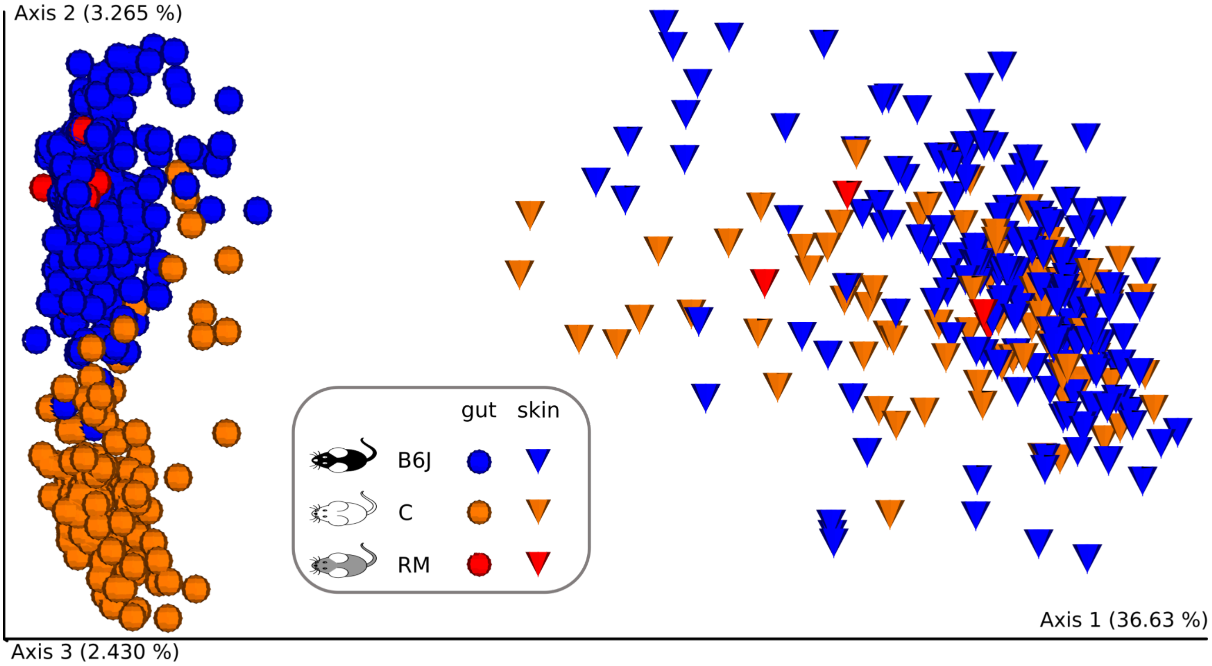

Figure S6: Differences in body sites. *PCoA of samples of the gut n=333 (spheres) and of the external skin of the left earlobe n=268 (cones) based on unweighted UniFrac shown in two clusters. The samples are additionally stratified by their host genotype showing B6J (blue), C (orange), and B6CF1 (red) mice, which form two clusters in the gut samples and one cluster in the skin samples.*

Figure S7

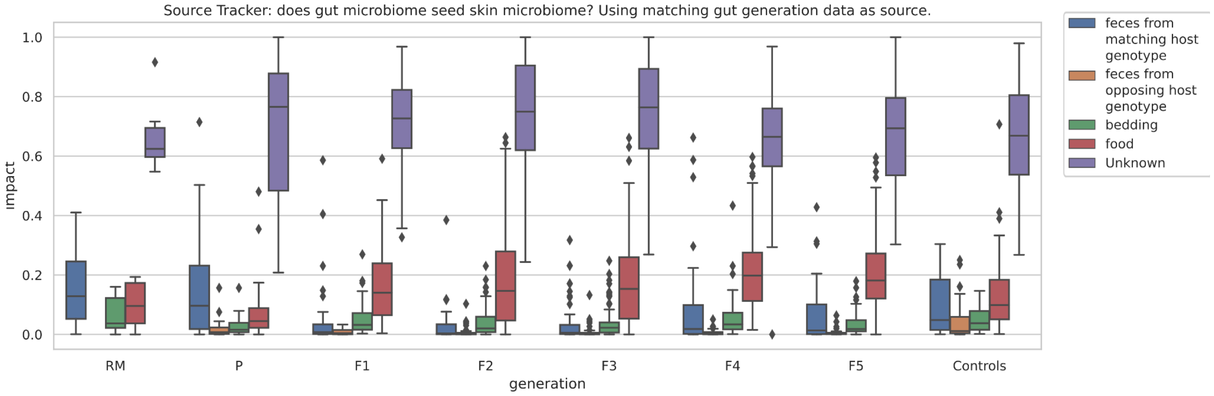

Figure S7: Impact of External Factors on the Skin Microbiome. *We used SourceTracker to investigate whether the external factors of bedding (green) or food (red) had an impact on the skin microbiome of the mice. Additionally, we included if mice were impacted by microbiota from feces from matching host genotype"host genotype" (blue) or from opposing host genotype"host genotype" (orange). For variables outside the assigned categories SourceTracker assigns the label Unknown (purple). This analysis was stratified by generations as well as adding the control mice.*

Figure S8

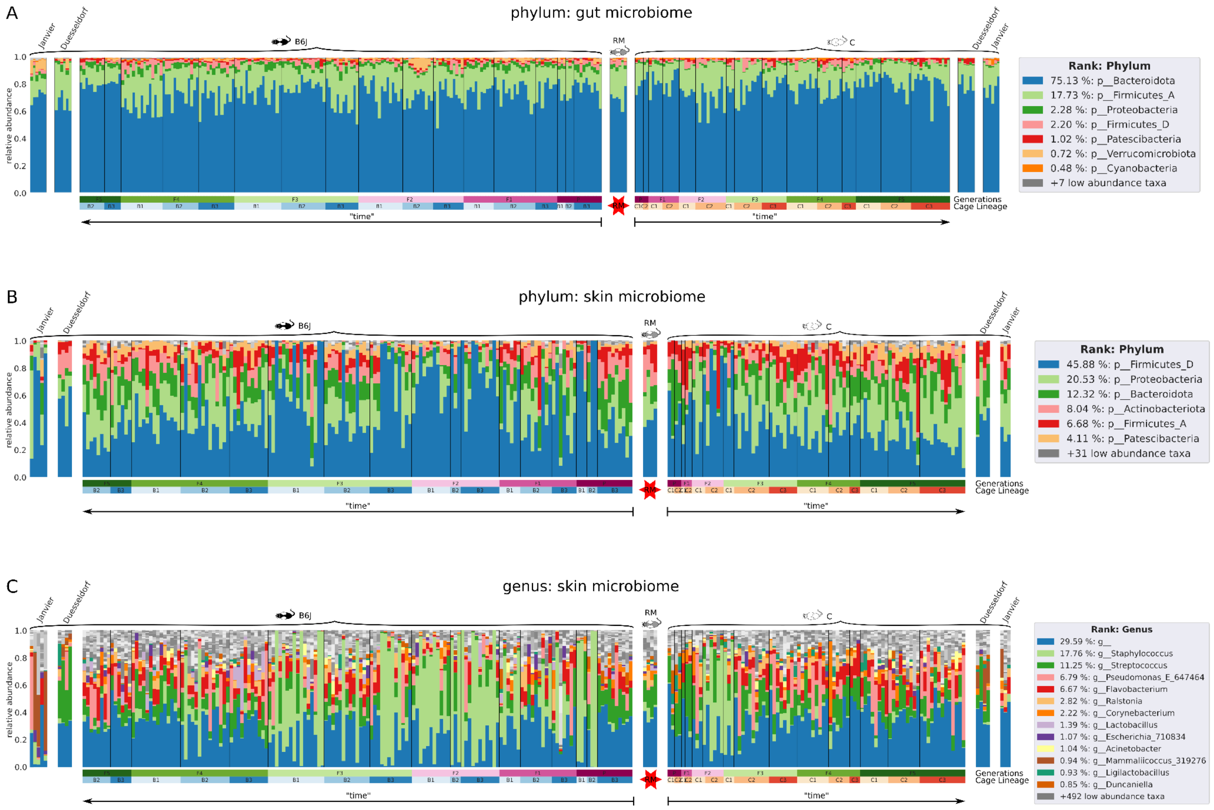

Figure S8: Taxonomy Barplots. *A) Phylum composition of 333 gut samples, rarefied to 6000 reads. Purple to green bar indicates generations, blueish and orange bar indicate cage lineage. Six samples of recipient mothers (RM) of hybrid host genotype"host genotype" B6CF1 (gray) are marked by the red star. Offspring generations (P to F5) are temporarily aligned to the left for B6J (black) and to the right for C (white), respectively; followed by six control samples each from mice obtained from another facility Duesseldorf (Germany) and ordered from Janvier (France). Cage lineages B1-B3 and C1-C3 are color coded in blue and orange, respectively. B) Phylum composition of 2628 external skin of the left earlobe samples, rarefied to 1000 reads. C) Same as B, but on Genus level.*

Figure S9

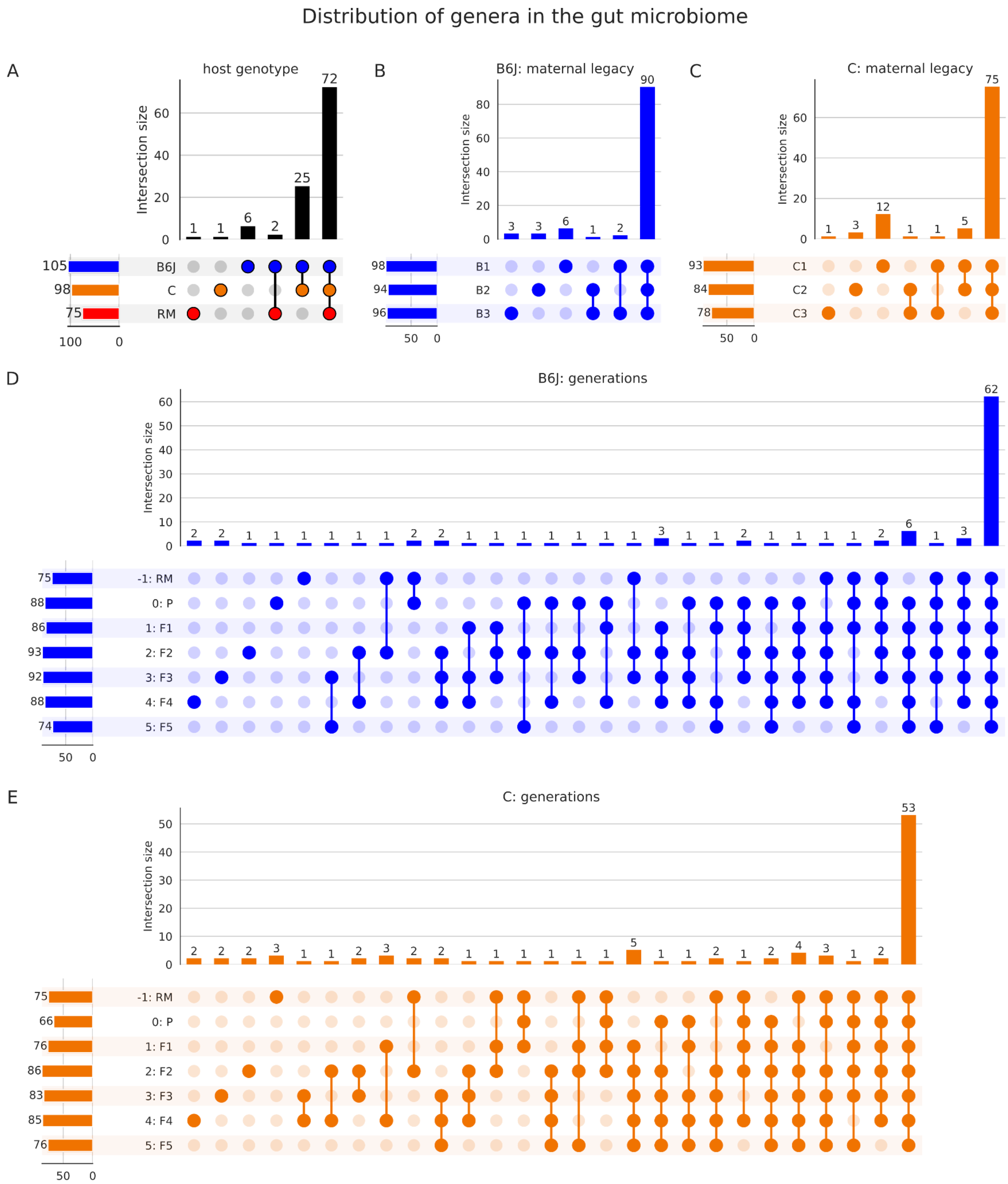

Figure S9: Distribution of Taxa in the gut. *To investigate the size of the core microbiome and overlapping taxa in the gut we created Upsetplots. The intersection size indicates how many taxa exist in a group, the circles show which categories the group consists of. The bars on the left sum up the total amount of taxa per category. A) Upsetplot comparing the "host genotypes" RM, C and B6J. B) This Upsetplot shows the distribution of taxa from the perspective of maternal legacy of the B6J mice. C) Analogous to Panel B but for C mice. D) This Upsetplot shows B6J and RM samples sorted by generation including RM. E) Analogous to Panel D but for C mice.*

Figure S10

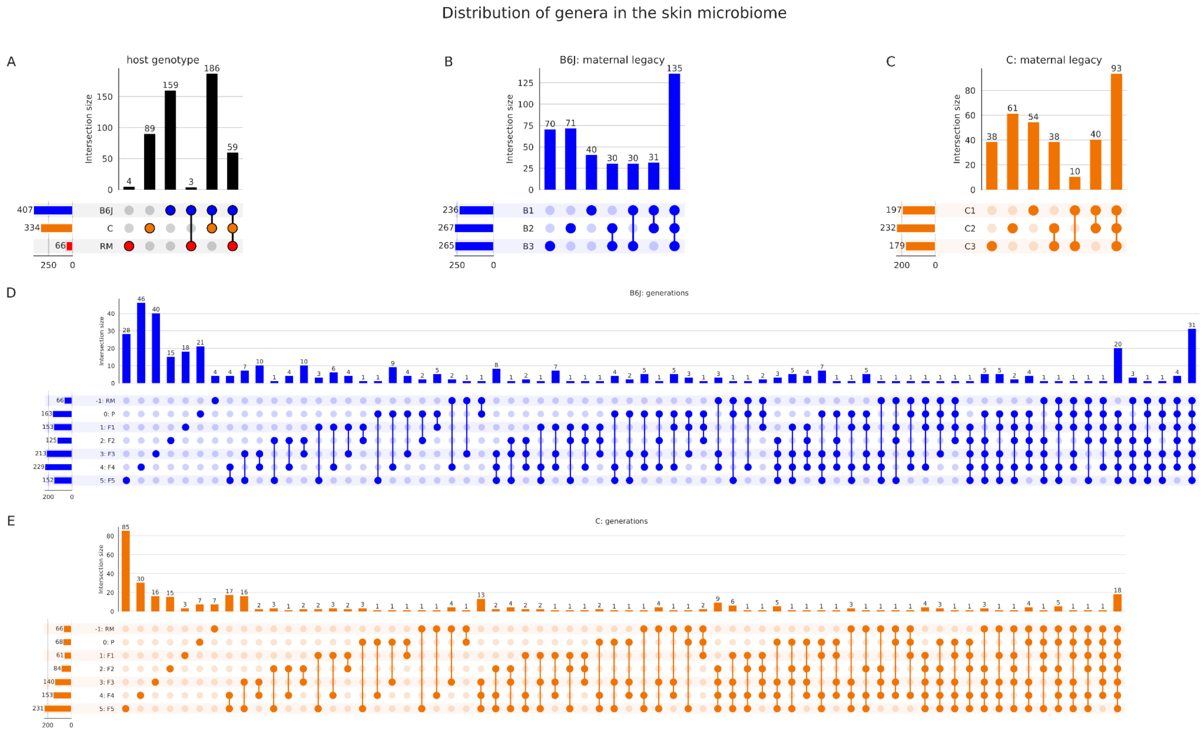

Figure S10: Distribution of Taxa in the Skin. *To investigate the size of the core microbiome and overlapping taxa in the skin we created Upsetplots. The intersection size indicates how many taxa exist in a group, the circles show which categories the group consists of. The bars on the left sum up the total amount of taxa per category. A) Upsetplot comparing the "host genotypes" RM, C and B6J. B) This Upsetplot shows the distribution of taxa from the perspective of "maternal legacy" of the B6J mice. C) Analogous to Panel B but for C mice. D) This Upsetplot shows B6J and RM samples sorted by generation including RM. E) Analogous to Panel D but for C mice.*
