# Supplementary material for: The host genotype actively shapes its microbiome across generations in laboratory mice
Source: Microbiome. 2024 Dec 5;12:256. doi: 10.1186/s40168-024-01954-2 (PMC11619136; doi:10.1186/s40168-024-01954-2)
Supplement: Supplementary file 30 [file 40168_2024_1954_MOESM2_ESM.zip › microbiome_benga_hostgenotype-1.2.0/Outdated_analyses/notes_benga.odt]

- It is discussed since years if and how microbes may have co-evolved with its hosts, to optimally fit the host’s needs. (Gutachten über Benga FoKo)

- The main factors that contribute to the inter-individual variation of the intestinal microbiota are the environment, diet, age, gender and genotype (Aus Laurentius 1stem FoKo Antrag und (Laukens et al., 2016))

- Whether and to which extent the host genotype shapes the gut microbiome is still subject of debate. While some allocate to the genotype a decisive role, others demonstrate that the foster mother gut microbiota rather than the genotype is essential in the future composition of the gut microbiota. (Aus Laurentius 1stem FoKo Antrag)

-  (Präsentation 2. FoKo Antrag)

- pro genotype:

  - It was shown by fingerprinting techniques, such as automated ribosomal intergenic spacer analysis (ARISA), that genotype is a stronger determinant than sex of the mouse gut microbiota (Kovacs et al., 2011) (erster FoKo Antrag)

  - It seems that the composition of the gut microbiota is genetically regulated among other undefined loci by the major histocompatibility complex (MHC), as demonstrated by the analyses of microbiome at the level of bacteria-derived cellular fatty acids (Toivanen et al., 2001). (erster FoKo Antrag)

  - Moreover, the breeder and genetic background influence the composition of gut murine microbiota (Ericsson et al., 2015) (erster FoKo Antrag)

  - a drift of the microbiota to a genotype and facility-specific composition, seem to occur under the influence of the local environmental factors and housing practices (Friswell et al., 2010) (erster FoKo Antrag)

  - Nevertheless, the functionality and integrity of the immune system contributes essentially to the distribution and abundance of the gut microbiota (Thoene-Reineke et al., 2014).

  - Moeller et al. 2018

    - 11 host generations!

    - two wild populations -> 17 inbred lines

    - "Individual- and population-level microbiota compositions were maintained within mouse lines throughout the experiment, indicating predominantly vertical inheritance of the microbiota"

    - Illumina V45 = 212 samples

    - "Comparing BSDDs within and between mouse lines indicated that vertical inheritance was the primary mode of gut bacterial transmission during the transition from the wild to the laboratory and subsequent inbreeding."

    - "the distinctiveness of Edmonton and Tucson microbiotas was still evident in the 10th generation of the experiment"

    - genetics (two wild strains) maintain microbiome through 11 generations in same environment but kept separate :-)

- pro Mother

  - Hansen et al., 2015, assumed that the foster mother gut microbiota rather than the genotype might influence the composition of the gut microbiota in the early life (Hansen et al., 2014b) (erster FoKo Antrag)

  - whereas Hildebrand et al., accounts for the genetics less than 20% of the gut microbiota variation (Hildebrand et al., 2013) (erster FoKo Antrag)

  - Friswell et al 2010:

    - "dominance of environmental influences on the GTM [= gastrointestinal tract microbiota]"

    - "probably due to difficulties associated with experimentally separating environmental from genetic influences."

    - embryo transfer 1 Mutter 3+3 Kinder, ABER nur P generation bepropt. PCR-DGGE V23 gut

  - Hansen et al 2014:

    - C-section from biological mothers, then cross-fostered with different genotype mothers

    - only P generation

    - fecal & ileum, 5/4 weeks of age, V3 DGGE AND 454/FLX Titanium (Roche) pyrosequencing of the V3 and V4 region of the 16S rRNA

    - "Thus, in these mice, it is clear that the early environment to which the newborn pups are introduced governs the gut microbiota composition later in life up to at least 5 wk of age, despite the genetic drive"

  - Hildebrand et al 2013

    - V35 via 454, Qiime 1.4

    - only cohousing 5 strains = 1 Generation, 10 replicates

  - Robertson et al. 2019

    - "Using reciprocal inter-crosses of P1 parents, we identify dissymmetry in F2 community structures caused by maternal transmission"

    - two lines of C57BL/6 mice (JAX & TAC)

    - "Our results establish that F2 littermates have greater homogeneity in microbiota than co-housed mice"

    - "specifically highlighted the importance of maternal transmission for Akkermansia"

    - SPF

    - --> vendor has sig. diff microbiomes for same strain

    - "Co-housing Does Not Normalize Mucosal Bacterial Community Structure" + "but that early life exposure and parental influences on community structure are preserved"

    - "we have demonstrated that variation in microbiota is negligible in F2-generation littermate mice"

    - --> environment (Jacson / Tac) is overridden after F2 generations :-)

  - Ericsson et al. 2015

    - V4 Illumina

    - two Vendors (Jackson, Harlan Sprague Dawley) x multiple strains (A/{J|NHsd}, BALB/c{J|NHsd}, C57BL/6{J|NHsd}), feces

    - time series, but just one generation

- Hypothese 2: "possible effects of sex and cage on the gut microbiome shaping will be analysed." (erster FoKo Antrag)

- The housing of the mice and the breeding procedures will occur into individually ventilated cages (IVCs), since complex microbiota seem to be protected into IVC housing system over long time (Lundberg et al., 2017) (erster FoKo Antrag)

- parental mice, which will be colonised naturally with the flora of their foster-mothers (erster FoKo Antrag)

Argumente zur Motivation

- Microbiome impacts phenotype

Meine Fragen:

- warum mehrere Generationen?

- are all 6 recipient mice sisters?

  - nein: "Ich glaube dass 3/6 waren sogar Wurf Geschwister." (Laurentiu)

Paper Fragen:

- ist Mikrobiom der forster mothers eine "echte" Mischung oder einem der beiden Genotypen ähnlicher?

"We herein define the metagenome as the sum of all host genes plus all organism genes of the microbiome" from Stappenbeck & Virgin, Nature 2016

Toll-like and NOD-like host receptors sense bacteria and thus shape microbiome: Friswell et al 2010

high environmental impact on newborns due to feces in bedding: Hansen at al. 2014

"It is widely recognized that independent lineages of animals, whether bred in the same facility or purchased from a vendor, display differences in community structure of the gut microbiota (Franklin and Ericsson, 2017)" from Robertson 2019

"Another approach

involves embryo transfers from the WT and KO strains into a foster dam, which can be effective if the microbiota is normalized across foster dams (e.g., using littermates), but involves coordinating timing and a potentially cost-limiting surgery." from Robertson 2019 --> NEIN ICH DENKE DAS GEHT NICHT

Existing literature is inconclusive about effect sizes on the assembly of the microbiome when transmitted to the next host generation. XXX favor dominance of the "maternal legacy", i.e. microbes transferred from the mother to her children via e.g. the passage through the birth canal, weaning, coprophagy or grooming. Others YYY postulate dominance of the offspring "host genetics", which translates to an active shaping of the child microbiome through e.g. its immune system. To complicate matters, environmental aspects like diet, bedding, level of isolation (SPF, CF, IVC, open cages) and behavioral differences like host stress will impact the microbiome in various levels.

With our following breeding experiment, we uncoupled "maternal legacy" from "host genetics" by obtaining 2-cell stage embryos of two representative genotypes C57BL6/J (n=42) and BALB/c (n=57) and transferring a mixture of both genotype embryos into six C57BL6/J (dam) | BALB/c (sire) hybrid recipient dam mice (RM). We subsequently tracked microbial composition of RM mice and through time along six inbreed generations, strictly separated by host genotype. We aimed to minimize environmental impact on the microbiome by housing all mice in a specific pathogen free facility with individually ventilated cages, identical diet and bedding from the same lot and sampling of colon content and skin of ear at an adult stage with fully developed microbiome at 15 weeks of age. Housing occurred at 12:12h light/dark cycles at 22±2°C room temperature and 55±5% humidity in weekly changed, autoclaved cages. For reference, we also sampled six mice of each genotype independently obtained from our mouse facility (Duesseldorf, Germany) with less strict SPF isolation, i.e. open cages, and bought from a commercial vendor (Janvier, France). To further dissect "maternal legacy", we implemented three cage lineages per genotype, i.e. strict inbreed lineages that never came in contact.

"Host genetics" overrides "maternal legacy".

Our microbial gut data show a very strong overriding impact of "host genetics" over "maternal legacy" for constant environments. Interestingly, this effect is not yet pronounced in the P generation, but in all that follow (Fig Ya: Kruskal-Wallis H-test on Faith's PD: p < 0.01, ns for P and F5 and green line in Fig Yb: Permanova with 999 permutations on weighted UniFrac: p < 0.002, ns for P).

The RM mice are generated by mating female C57BL6/J with male BALB/c. The relatively high microbial similarity at the P generation (no significant difference, see above) seems to favor the "maternal legacy" effect; the weighted UniFrac distance between genotypes (green line in Fig Yb) is lower than the average distance between any pair of mice from different cages within the same cage lineage (and thus genotype, brown dotted line). In accordance, the RM microbiome seems to be more similar to C57BL6/J Duesseldorf controls than to BALB/c Duesseldorf controls (Fig Yc, Kruskal-Wallis H-test on weighted UniFrac: p=0.053). However, the microbiome between RM and mice of the P generation is significantly different (Kruskal-Wallis H-test on weighted UniFrac: p < 0.01), which might result from the relatively invasive embryo transplantation with preceding skin disinfection as an environmental distortion. This would explain the drop in alpha diversity, although only borderline significant (Kruskal-Wallis H-test on Faith's PD: p=0.062). To explain the significant microbial dissociation in all following generations (F1 to F5) though, we must favor "host genetics" over "maternal legacy", especially since microbiomes started quite homogeneous in the P generation, but drifted apart from F1 onwards, while we kept environmental changes minimal. Considering the relatively constant trajectory of microbial differences between C57BL6/J and RM and the continuously increasing difference between BALB/c and RM (Fig Yb, blue and orange lines, respectively) emphasizes that the microbiome of the hybrid RM mice is dominantly that of C57BL6/J mice and there must be an active shaping in the BALB/c animals.

Despite the clear dominance of "host genetics", environmental aspects easily exceeds this effect, as can be seen by the significantly higher alpha diversity (Fig Ya, Kruskal-Wallis H-test on Faith's PD: p < 0.01) of Janvier control mice, which were bought from a commercial vendor and only acclimatized for one week in our local facility prior to sampling at 15 weeks of age and the generally larger beta diversity distance of mice from our breeding experiment with Janvier controls, compared to Duesseldorf controls (Fig Yc: dashed vs solid lines, respectively). The environment is probably furthermore a limiting factor for the degree of genotype specific tailoring of the microbiome. Our narrow environment (autoclaved cages, autoclaved rodent chow and autoclaved  bedding, acidified water, individually ventilated cages) provided a restricted set of microbes the hosts could source from, such that genotype differences peek around F2 and probably shows intergenerational cycling thereafter (Referenz von Laurentiu?). This would explain the flips in distance of BALB/c to control mice (orange lines in Fig Yc), whereas distances of C57BL6/J to control mice remain relatively stable as only the BALB/c "host genetics" actively tailors its microbiome away from the shared starting point, which is already C57BL6/J-like, in the P generation. 

"Maternal legacy" is not negligible.

To better quantify "host genetics" vs "maternal legacy" we established three cage lineages per host genotype. To amplify "host genetics", all animals in the BALB/c cage lineages decent from a single P male; compared to C57BL6/J, where each cage lineage decent from a separate P male. Furthermore, establishment of the third cage lineage for BALB/c was deferred until F3.

Comparing beta diversity and quantifying the impact of microbiome shaping via source tracker analysis (Fig Yd + Fig Supp A) shows significantly higher similarity between mice and their true biological/genetic dams (blue and orange boxes) than mice with dams in other cage lineages of the same genotype (gray boxes; two-sided Mann-Whitney-Wilcoxon tests with Benjamini-Hochberg correction on unweighted UniFrac) in all but the P generation stratified by genotype to keep "host genetics" constant. This clearly indicates existence of the "maternal legacy". Although, "host genetics" is even stronger (as presented previously) and by comparing distances to dams of the opposite genotype (green boxes in Fig Yd; two-sided Mann-Whitney-Wilcoxon tests with Benjamini-Hochberg correction on unweighted UniFrac) for most generations / genotypes. Despite our moderate strategy to increase "host genetics" in the BALB/c cage lineage, the unweighted UniFrac distances between true and other dams is not significantly larger than in the C57BL6/J cage lineage (two-sided Mann-Whitney-Wilcoxon test, "stacking" distances of gray boxes in Fig Yd).

"Host genetics" / "maternal legacy" homogenizes the gut microbiome more efficiently than co-housing.
Complementary to our breeding experiment, in which we controlled "maternal legacy" and varied "host genetics", Robertsen et al. did the opposite with an experiment in 2019, where they obtained mice of the same genotype from two different vendors: C57/Bl6J from Jackson Laboratories (JAX) and C57/Bl6N from Taconic Farms (TAC). They found, that "maternal legacy" better standardizes the gut microbiome (ileum, colon, feces) within two generations of littermates than co-housing first generation offspring. Their published raw sequence reads allowed us to conduct a meta analysis, see Figure Ye. In the following, we omit Robertsen's ileum and feces samples, as we did not sequence these body sites.

genotype

Emphasizing the strong impact of "host genetics", our C57BL6/J gut microbial samples (n=201: generations P to F5 and controls) are in fact significantly more similar to Robertsen's C57/Bl6 parental and littermate mice of both vendors (n=28 TAC + n=27 JAX) than our BALB/c samples (n=126: generations P to F5 and controls; two sided Mann-Whitney-Wilcoxon test with Benjamini-Hochberg correction).

vendor

In addition, "maternal legacy" seems to prevail across different experimentalists, sampling modalities and facilities, as our C57BL6/J samples (n=201) are significantly closer to Robertsen's JAX (n=27) than to Robertsen's TAC (n=28) samples - our mice colony originates from JAX. Since Robertsen et al. did unfortunately not fully uncouple "host genetics" from "maternal legacy" via embryo transfer through a dam of different genotype, one cannot completely rule out "host genetics" effects, as genotype of mice from JAX and TAC might differ more than assumed. On the other hand, hybridization in Robertsen's littermate experiment retained a significantly different microbiome between offspring of JAX sire and TAC dam mouse vs. JAX dam and TAC sire mouse, although genotype should have recombined identically in F1 and F2 generations (data not reproduced here, cf. Robertsen et al. 2019).

littermates

We assume relatively high environmental differences for Robertsen's P1 mice, as they are directly sampled one week after purchase from JAX and TAC. The F1 and F2 mice for Robertsen's littermates experiment are housed under a much more constant environment: SPF at the Division of Comparative Medicine of the University of Toronto, filter-top cages, commercial pelleted food and non-acidified water. Stratifying Robertsen's 27 JAX mice (but not the 28 TAC mice) by generations, shows that they indeed become significantly more similar to our n=201 C57BL6/J mice over time - in accordance with our previous observations.

This active microbiome tailoring can only incompletely be achieved via co-housing, as previously concluded by Robertsen et al. There is a significant increase in similarity of microbial communities between our C57BL6/J samples (n=201) and co-housed JAX (n=16) and TAC (n=16) F1 mice in Robertsen's experiment, compared to non co-housed JAX (n=12) and TAC (n=12) mice. However, the increase in similarity to our C57BL6/J samples (n=201) is significantly stronger in Robertsen's F2 littermate JAX (n=8) mice.

generations

Despite marked biological (sampling at 15 vs. 8 weeks of age, acidified vs. non acidified water, cages changed weekly vs. bi-monthly, commercial pelleted food vs. autoclaved chow) and technical (different technicians, V34 vs. V4 16S rRNA target region, different sequencing centers) differences between Robertsen's and our microbiome profiling, it is interesting to see, that our C57BL6/J samples become significantly more similar (except F3, F5) to Robertsen's F2 JAX samples with proceeding generations. This might point to a universal genotype specific core microbiome and warrants further investigation. Due to different used 16S rRNA target regions, we assume incompatible taxonomic assignments (cf tremendous shifts in Bacteroidota / Firmicutes_A ratio in Suppl Fig Z) and therefore refrain from further investigations on taxonomic features.

for discussion

Previous work to disentangle the impact of "host genetics" vs. "maternal legacy" on the composition of offspring microbiome only sampled the first offspring generation (Friswell et al. 2010, Hansen et al. 2014, Hildebrand et al. 2013, Ericsson et al. 2015). Since no significant microbial difference could be detected after embryo transfer, cross-fostering or co-housing, exactly as in the microbial profiles in our P generation, authors rightfully concluded that "maternal legacy" dominates any "host genetics" effects, if present at all. However, this is based on a quite incomplete observation. Our data clearly show that microbial differences manifests in the next generation and further increase over time, at least for constant environments. We therefore argue that our data is compatible with previous findings, however our longer temporal sampling suggests the opposite conclusion, namely that "host genetics" dominates "maternal legacy", which for itself but to a lesser extend is also acting in tailoring the microbiome.

Skin microbiome shows effects of "host genetics" but lacks "maternal legacy".

We sampled the skin of the left earlobe of all mice in addition to the previously discussed colon samples. Lower biomass led to fewer reads and subsequently more samples were lost through quality control, invalidating application of statistical tests due to low sample numbers for some of the following comparisons.

As in the gut, "host genetics" shapes the skin microbial communities in a genotype dependent manner although to a milder extend (Fig suppYb; significantly different unweighted UniFrac distances between genotypes, tested via PERMANOVA with 999 permutations in F3 and F5, P and F1 have too few BALB/c samples, F2's p-value of 0.076 is close but does not reach significance niveau). The clear split between genotypes in the gut samples is not recapitulated for skin. The RM skin microbiome flips being more similar to C57BL6/J or to BALB/c three times throughout the P to F5 generations. Using control mice as reference instead (Fig suppYc), the skin microbiome seems to be more similar to C57BL6/J for all but the P generation. Interestingly, alpha diversity (measured as Faith's PD, Fig supp Ya) is never significantly different across any generation; this is also true for the alternative metrics "Shannon diversity" and "observed features", i.e. the raw number of different ASVs.

We were not able to measure a "maternal legacy" effect in the skin samples, neither by comparing beta diversity (Fig suppYd) nor by source tracking (Fig suppYe). Again, low sample numbers prohibit statistical testing, but microbial alpha diversity between Janvier and Duesseldorf controls seem to be markedly different (Fig supp Ya), pointing to a stronger "environmental" impact on microbial composition.

Weather or not the "host genetics" differences in the skin microbiome of the earlobe are actively tailored by the mice or is an indirect reflection of the active tailoring of the gut microbiome with subsequent spreading of these microbes to their skin cannot be unambiguously answered from our data. The interactions between host immune system and skin is presumed to be much less intensive. External skin is in general more prone to environmental conditions and thus much harder to control for. 

Using five chow and five bedding control samples of the lots used for mice housing in addition to gut microbiome samples, stratified by genotype and generation as "sources", we quantified the contribution of community assembly in the skin ("sink") via source tracking (Fig suppB). Note that different read coverage between sinks and sources likely skews results. The source tracking analysis shows that skin of earlobe microbial community is only composed by 6% on average of microbes found in genotype matching gut samples of the same generation. Microbes from the "opposite" gut microbiome accounts for negligible 1% on average. Cage bedding material (5%) and mice chow (18%) had similar or approx. three-fold stronger impact on skin microbiome assembly, whereas the huge majority of community composition remains unknown (70%). We conclude that environmental effects dominate the skin microbiome with clear imprinting of "host genetics" tailoring but no detectable "maternal legacy" effects.

Supp. Fig. Z

Supp Fig A

Supp Fig B

new Figure 7
